# Multi-network dynamical structure of the human brain in the setting of chronic pain: a coordinate-based meta-analysis

**DOI:** 10.1093/braincomms/fcaf343

**Published:** 2025-10-29

**Authors:** Vukshitha Dhanaraj, Nathaniel W Rolfe, Nicholas B Dadario, Jasneet Dhaliwal, Nardin Samuel, Jorge Hormovas, Isabella M Young, Charles A Odonkor, Jacky Yeung, Charles Teo, Stephane Doyen, Michael E Sughrue

**Affiliations:** Centre for Minimally Invasive Neurosurgery, Prince of Wales Private Hospital, Suite 19, Level 7 Prince of Wales Private Hospital, Randwick, Sydney, NSW 2031, Australia; Department of Neurological Surgery, Columbia University Irving Medical Center/NY-Presbyterian Hospital, New York, NY 10032, USA; Robert Wood Johnson Medical School, Rutgers University, New Brunswick, NJ 08901, USA; UCL Medical School, University College London, London WC1E 6BT, United Kingdom; Division of Neurosurgery, Department of Surgery, University of Toronto, Toronto Canada M5G1X5; Centre for Minimally Invasive Neurosurgery, Prince of Wales Private Hospital, Suite 19, Level 7 Prince of Wales Private Hospital, Randwick, Sydney, NSW 2031, Australia; Department of Neurosurgery, Omniscient Neurotechnology, Sydney, NSW 2000, Australia; Department of Neurosurgery, Yale University School of Medicine, New Haven, CT, USA; Department of Neurosurgery, Yale University School of Medicine, New Haven, CT, USA; Centre for Minimally Invasive Neurosurgery, Prince of Wales Private Hospital, Suite 19, Level 7 Prince of Wales Private Hospital, Randwick, Sydney, NSW 2031, Australia; Department of Neurosurgery, Omniscient Neurotechnology, Sydney, NSW 2000, Australia; Department of Neurological Surgery, Columbia University Irving Medical Center/NY-Presbyterian Hospital, New York, NY 10032, USA

**Keywords:** fMRI, connectomics, chronic pain

## Abstract

The treatment of chronic pain represents a widespread clinical challenge. Current approaches to network-based mapping of the cerebral cortex have the potential to localize chronic pain in the brain. In an effort to further characterize the dynamical brain networks, or the ‘dynome’ in the setting of chronic pain, we performed a Coordinate-Based Meta-Analysis of resting-state functional Magnetic Resonance Imaging studies on chronic pain to create a multinetwork dynome of chronic pain. A cluster-level analysis generated seven statistically significant activation likelihood estimates (ALEs): one for chronic pain as a whole dynome, three for chronic pain conditions, and three for chronic pain mechanisms. Chronic pain is a complex disease process involving tripartite network dysfunction encompassing the Default Mode Network, Central Executive Network and Salience Network. Chronic visceral pain was distinct from chronic headache and chronic musculoskeletal pain, and chronic pain mechanisms have the potential to share common cortical network rearrangements with their respective chronic pain conditions. Collectively, this work represents the first anatomically specific network-based cortical map of chronic pain, with representation of disease-specific and mechanism-specific disruptions in cortical function.

## Teaser

Dhanaraj *et al*. report that chronic pain involves tripartite network dysfunction encompassing the Default Mode Network, Central Executive Network, and the Salience Network. Their Coordinate-Based Meta-Analysis of resting-state functional MRI studies provides an anatomically specific network-based cortical map of chronic pain, highlighting disruptions in cortical function for different pain conditions.

## Graphical Abstract

**Graphical Abstract:**
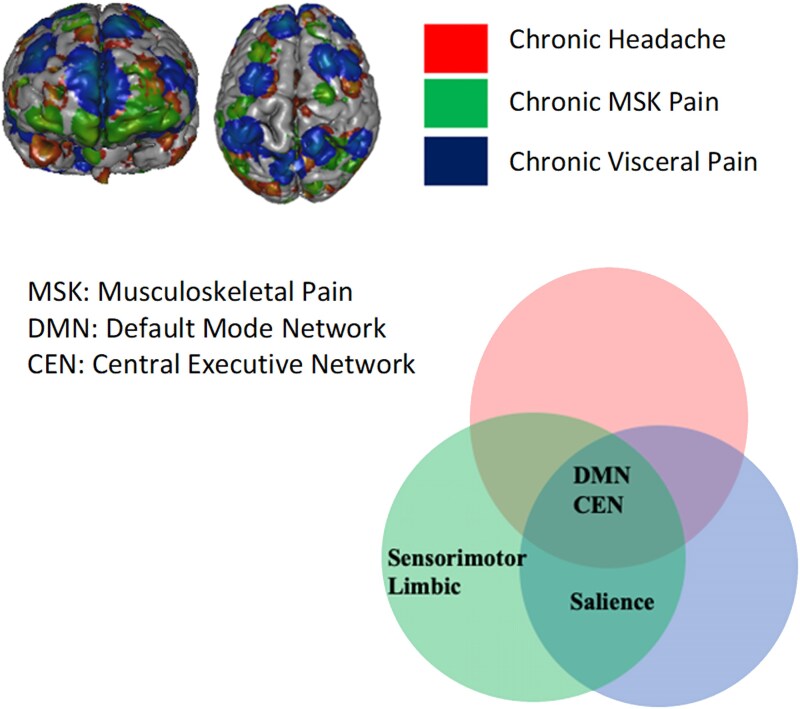

## Introduction

Chronic pain represents a complex, multi-dimensional experience resulting from the interplay of sensory, emotional, and cognitive processes that reinforce one another during the development of chronic pain.^1,2^ These interactions subsequently manifest as spontaneous pain, hyperalgesia (exaggerated reactions to noxious stimuli), allodynia (pain from innocuous stimuli) or secondary hyperalgesia (pain extending beyond primary injury).^3,4^ The International Association for the Study of Pain (IASP) defines chronic pain as ‘persistent or recurrent pain lasting or recurring for longer than 3 months’.^5^ Unfortunately, despite growing research into treatments for chronic pain, both conventional^3,6^ and non-conventional^7^ therapies show limited efficacy. This therapeutic gap suggests that our understanding of the diverse pathophysiologic mechanisms underlying chronic pain conditions is limited.

Significant advancements in neuroimaging technologies have improved our understanding of pain processing and our ability to localize chronic pain.^8^ In particular, functional Magnetic Resonance Imaging (fMRI) has established distinct neural differences between chronic pain patients and healthy individuals.^9-11^ Task-based studies have formed the foundation of fMRI research in chronic pain, where sensory-discriminative,^12^ emotional-modulatory,^13^ and cognitive-affective^14^ paradigms assess Blood-Oxygenation Level-Dependent (BOLD) signal changes from baseline. However, this explicit manipulation of an individual’s pain-attentional state involves the supposition that individuals have an unvarying ‘normal’ baseline neurocognitive state that is only altered when manipulated externally. By contrast, neural activity across the brain is dynamic and fluctuates spontaneously, and therefore vacillates even in the absence of unchanging stimuli.^15^ Resting-state fMRI (rs-fMRI) has emerged as a powerful tool to explore this intrinsic, fluctuating activity in the brain and offers deeper insights into attentional shifts and their impact on the subjective experience of chronic pain.^16,17^

Connectomics allows us to study connections within the nervous system, arranged as networks comprised of ‘nodes’ and ‘edges’, with nodes being anatomical regions of the brain and edges the functional connections between them.^18^ Resting-state networks are intrinsic regions of the brain that demonstrate functional connectivity and display a specific spatiotemporal arrangement. Neuroimaging-based analyses provide a powerful tool to identify altered neural signals of resting-state networks in chronic pain patients compared to healthy controls.^19-23^ However, isolated studies have stark cerebral inconsistencies, are susceptible to false positives,^24,25^ are underpowered,^26^ and vary in experimental methodology.^27^ Furthermore, the sparsity of replication studies further complicates the ability to confidently localize chronic pain.^28^

Coordinate-based meta-analyses (CBMA) are an analytic approach to capitalize on available neuroimaging research to quantitatively represent the peak location of concurrently functionally active regions in the brain by analysing statistical data.^29^ Several task-based CBMAs have been conducted on acute pain in healthy volunteers and chronic pain in patients,^30-34^ highlighting potential circuitry functionally involved in the brain’s perception of pain. However, these analyses were subject to their own problems, with multiple studies not finding any statistically conclusive results due to methodological differences.

Recent studies have sought to elucidate brain networks and characterize the type of dynamic interactions that occur between networks, a framework termed the dynome.^18^ We have applied the dynome framework to this study in order to better understand the dynamic brain rhythms involved in chronic pain states. As such, in the present study, we conduct the first CBMA of chronic pain using resting-state connectome studies. Our aim is to create an anatomically concise and comprehensive multinetwork ‘dynome’ model for the core neurophysiologic basis of chronic pain.

## Methods

We conducted a systematic review and meta-analysis that followed the Preferred Reporting Items for Systematic Reviews and Meta-analyses (PRISMA) reporting guidelines^35^ and well-known field-standard guidelines for CBMA.^29^ This study did not require review board approval as anonymous statistics from published data were utilized. The review was not registered and did not require a protocol.

### Paradigm and contrast

Our CBMA exclusively focused on rs-fMRI studies and therefore had no paradigm limitations, as the studies included were not task-based. This was standardized by confirming that all studies had clear instructions to evoke an explicit resting-state.^36,37^ This study only focused on the brain networks and anatomical parcellations that were underactive in chronic pain patients in comparison to healthy controls, reflected in the contrast: Healthy Controls > Chronic Pain Patients (HC > CPP). We plan on analysing overactive regions in a future study: Healthy Controls < Chronic Pain Patients (HC < CPP).

### Literature search

A preliminary literature search was conducted by two authors (V.D. and N.D.) on standard literature databases including PubMed, Embase, Web of Science, Scopus, and Google Scholar using the following search algorithm for studies published between January 1, 1990, to August 30, 2021: (fMRI OR ‘functional magnetic resonance imaging’ OR BOLD OR ‘Blood oxygenation level-dependent’) AND (‘chronic pain’ OR ‘pathological pain’ OR ‘neuropathic pain’ OR allodynia OR hyperalgesia). Existing fMRI data registers, including Sleuth 3.0.4^38^ and Neurosynth,^39^ were also utilized. Citation searching of retrieved primary studies was also conducted to maximize the breadth of candidate studies.

### Screening and inclusion and exclusion criteria

**Figure 1:**
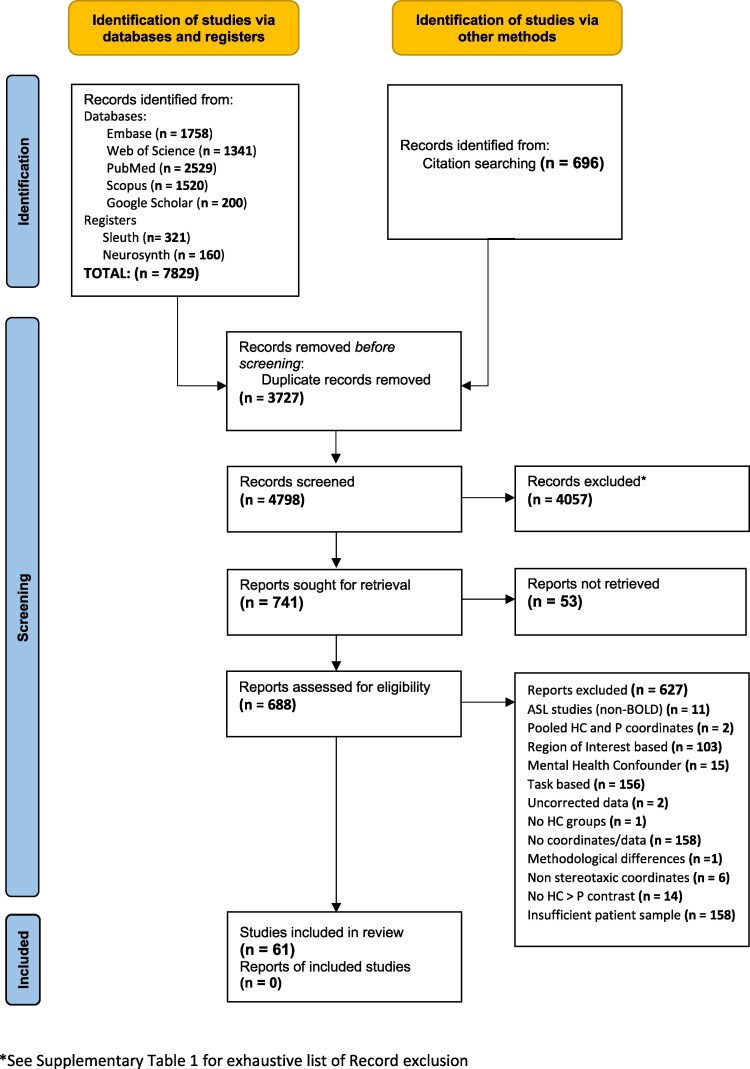
**PRISMA guidelines.** Diagram of the Study Selection Process conducted for the Systemic Review and Meta-analysis as per PRISMA guidelines. ASL, arterial spin labelling; Non-BOLD, blood-oxygenation-level dependent; HC, healthy controls; P, patient.

Title and abstract screening provided relevant articles for full-text screening. Supplementary Table 1 gives a comprehensive list of excluded reports. Full-text screening was subsequently conducted on selected studies using the generated Inclusion/Exclusion Criteria, which were consistent with CBMA guidelines (Table 1).^29^ The PRISMA flow chart illustrates the screening process (Fig. 1). The number of excluded articles and their reason for exclusion are summarized in Supplementary Table 1.

**Table 1:** Inclusion and exclusion criteria for the coordinate-based meta-analysis

Inclusion criteria	Exclusion criteria
Results presented in standard stereotaxic reference space coordinate system (MNI or Talairach coordinates)	Results presented in non-standard stereotaxic reference space coordinate system
Resting-state fMRI studies on chronic pain	Task-based fMRI studies on chronic pain
Brain responses monitored by fMRI Resting-State BOLD responses	Brain responses monitored through Arterial Spin Labelling Responses and other imaging methods such as Positron Emission Tomography (PET)
Whole Brain field of view to minimize bias towards priori Regions of Interest (ROI)	Seed-Based/ROI analyses
Chronic pain patients required formal diagnosis of chronic pain by a clinician or self-reported pain for more than 3 months as per the ICD-11^5^ criteria	Individuals that do not meet the chronic pain criteria
Peer-reviewed studies written in the English Language	Non-peer-reviewed studies not written in the English Language
Studies that included only adult populations in patient and control groups (>18 years)	Studies that included non-adult populations in patient and control groups (<18 years)
Sample size of >10 patients and >10 healthy controls in experiments	Sample size of <10 patients and <10 healthy controls in experiments
Results corrected for multiple comparisons by reporting foci at a voxel level threshold of *P*-value < 0.05 (uncorrected) or a corrected cluster probability of *P*-value < 0.05	Studies with inadequate statistical thresholds about their multiple comparison’s correction methods increasing false-positive rates
Experiments containing both chronic pain patient groups and healthy control groups.	Experiments with either chronic pain patient groups or healthy control groups.
Coordinates that were within-experiment and between-participants to minimize external and experimental confounders (e.g. different imaging procedures, variability in patient instructions)	Coordinates that were between-experiment and were within-participants increasing likelihood of external and experimental confounders
Studies that had coordinates for the contrast: Healthy Controls > Chronic Pain Patients (HC > CPP)	Studies that did not have coordinates for the contrast: Healthy Controls > Chronic Pain Patients (HC > CPP)

MNI, Montreal Neuroimaging Institute; fMRI, functional magnetic resonance imaging; BOLD: blood-oxygen level dependent; ICD-11, International Classification of Diseases-11.

### Data extraction

Between-participant (HC > CPP) Montreal Neuroimaging Institute (MNI) or Talairach coordinates were manually extracted from included studies. If studies had multiple contrasts representing underactive states, all contrasts were presented under one experiment to avoid overrepresentation. Information from studies was also collected, including chronic pain diagnosis, sample size, type of stereotaxic coordinates (MNI/Talairach), medication status, pain duration, and study methodology.

### Statistical analysis—generation of activation likelihood estimation

Our CBMA used the Activation Likelihood Estimation (ALE) methodology with a revised algorithm that allows for random effects interference.^40,41^ ALE models the likely convergence of foci across experiments and finds convergence patterns that are higher than the null distribution of random spatial associations. The foci act as epicentres for 3D Gaussian probability distributions, which represent spatial uncertainty related to the intrinsic limitations of fMRI methodology. Likelihoods of activation foci for experiments were pooled for each of the voxels across the meta-analysis, resulting in the formation of a modelled activation map, which combines to provide a corresponding voxel-based ALE score. Subsequent conjunction analysis reflects patterns of shared activation, allowing the construction of chronic pain maps.^40,41^ BrainMap Ginger ALE 2.3.6 was used to create ALEs from the extracted MNI coordinates.^40-42^ Differences in stereotaxic reference between studies were corrected for by converting all Talairach coordinates into the MNI coordinates using the icbm2tal transform statistical parametric mapping Conversion in GingerALE.^43,44^ Using a familywise corrected cluster-level significance of *P* < 0.05, and voxel-wise threshold permutation of *P* < 0.05, a single study analysis using cluster-level inference was performed in the MNI coordinate space. The Multi-image Analysis GUI (Mango) 4.0.1 was then used to overlay the ALE coordinate data over an MNI-normalized brain image for illustrative purposes.^45,46^

### Generation of sub-analyses

**Figure 2:**
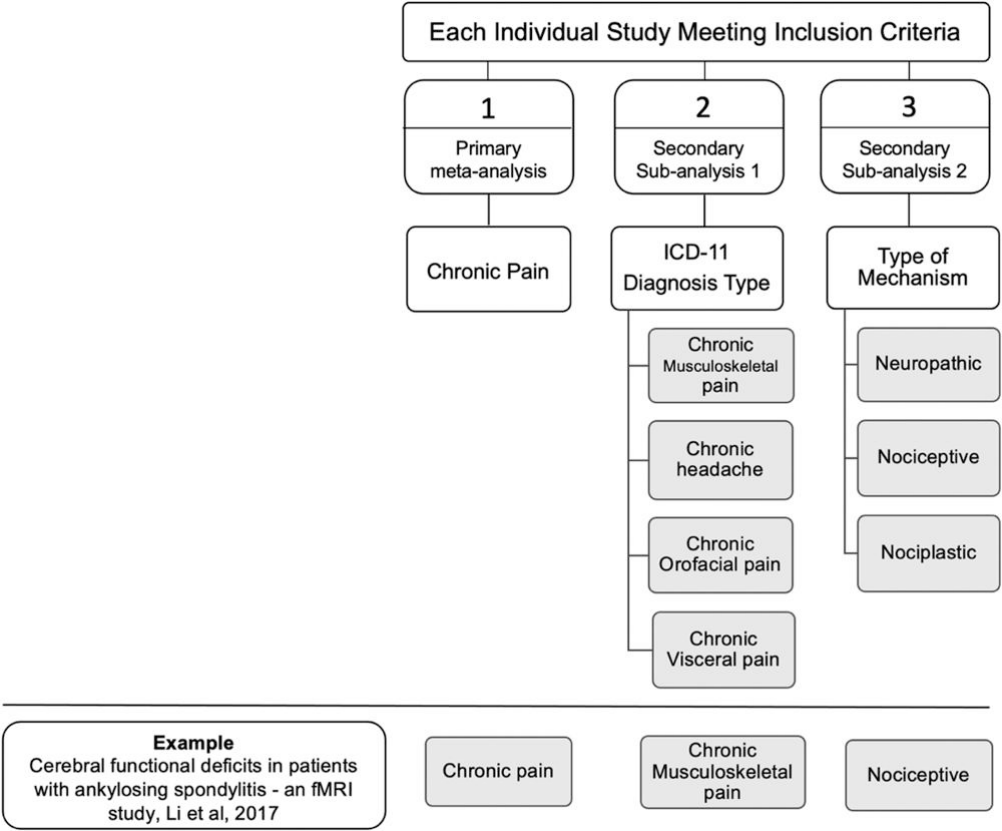
**Flow chart illustrating the meta-analysis generation process.** Each individual study meeting the inclusion criteria was allocated under one subcategory under each sub-analysis. Based on this, each study contributed to one primary meta-analysis and two sub-analyses. (There were exemptions when studies did not meet the criteria for a subcategory.) The example study by Li *et al*.^51^ provides an insight into the categorization process, where the study on chronic back pain (ankylosing spondylosis) was classified under (1) Chronic Pain, (2) Chronic Musculoskeletal pain, and (3) Nociceptive Pain subcategories under each sub-analysis. The same process was repeated for each study included. ICD-11, International Classification of Diseases 11; fMRI, functional Magnetic Resonance Imaging.

Included studies were combined to generate our primary meta-analysis: The Chronic Pain Dynome. Second, we generated two secondary sub-analyses using eminent taxonomy for chronic pain: Chronic Pain Conditions and Mechanisms of Chronic Pain. Supplementary Table 2 summarizes pain conditions captured by our meta-analysis. To categorize types of chronic pain conditions in the most systematic, reliable manner that carries clinical significance, the International Classification of Diseases-11 (ICD-11)^5^ scheme was used, and four major subcategories were derived: Chronic Musculoskeletal (MSK) pain, chronic headache, chronic orofacial pain, and chronic visceral pain. To classify studies based on mechanisms of chronic pain, published reviews were used.^47-50^ Based on these reviews, we classified the pain mechanism as: Neuropathic, Nociceptive, and Nociplastic Pain. Table 2 provides diagnostic definitions for the pain conditions and classified mechanisms. The two sub-analyses helped maximize the contribution of collected studies and subsequently helped create ALEs with sufficient power. Figure 2 is an illustration of how we generated the meta-analysis and sub-analyses.

**Table 2:** Clinical definitions for chronic pain conditions and mechanisms

Chronic MSK pain	Chronic pain that arises from musculoskeletal structures including joints and bones.^52^
Chronic Headache	Chronic headache diagnosis involves persistent headaches for 15 days or more lasting for more than 3 months^53^
Chronic Visceral Pain	Chronic visceral pain includes persistent or recurrent pain which is often poorly localized to a single internal organ ^54^
Nociceptive Pain	Pain that results from neural pathway activity as a consequence of ongoing inflammation, tissue damage, or potentially damaging stimuli^50^
Neuropathic Pain	Pain as a consequence of damage or disease of the somatosensory nervous system^55^
Nociplastic Pain	Maladaptive processes that impact nociceptive processing and modulation even in the absence of objective nerve or tissue damage^47^

Chronic MSK Pain: chronic musculoskeletal pain.

### Conversion of ALEs to Human Connectome Project parcellations

A previously published Human Connectome Project (HCP) parcellation map^56^ was overlapped on the constructed ALEs to match ALEs to their corresponding parcellations through a pre-set code on Python. The HCP expands Brodmann’s 47 Cortical regions to 180 intricate cortical parcellations, providing a superior level of resolution and a shared nomenclature for studying brain function and structure.^56,57^

### Conversion of HCP parcellations to networks

**Figure 3:**
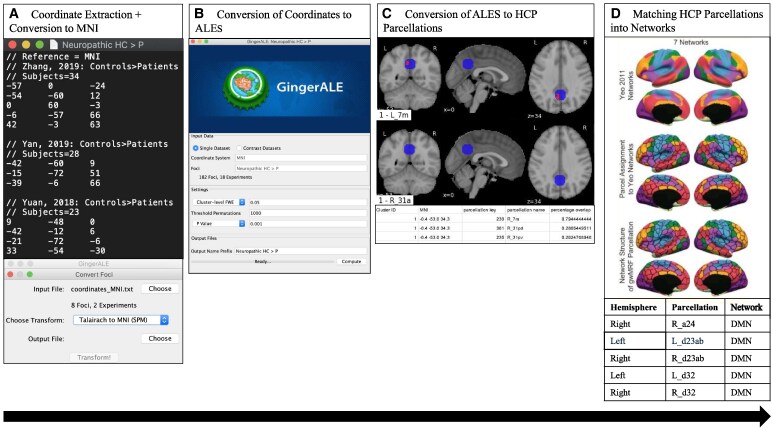
**Illustration of key components of the methodology.** (**A**) Coordinates corresponding to the contrast: Healthy Controls > Chronic Pain Patients (HC > CPP) were manually extracted from included studies and documented as text files. Stereotaxic coordinates that were in Talairach form were converted to MNI coordinate space using icbm2tal transform statistical parametric mapping Conversion in GingerALE.^43,44^ (**B**) An image of BrainMap Ginger ALE 2.3.6 software that was used to create ALEs from the extracted MNI coordinates, which were fed as text files into the software.^41,42^ A familywise corrected cluster-level significance of *P* < 0.05, and voxel-wise threshold permutation of *P* < 0.05 was set for cluster-level interference analysis on the MNI coordinate space. (**C**) Previously published HCP parcellation map^56^ was overlapped on the constructed ALEs to match ALEs to their corresponding parcellations. Two-dimensional images were created to visually represent the parcellations. The associated table provides the centred MNI coordinates corresponding to the parcellations. (**D**) The parcellations that were obtained were then matched to the core affiliate network based on Yeo’s 7-network model of the human cerebral cortex.^58^  Supplementary Documents 1 and 2 provide statistical data obtained for the seven sub-analyses used in the study and the Python code used. MNI, Montreal Neurological Institute; ALE, Activation Likelihood Estimation; HCP, Human Connectome Project; DMN, Default Mode Network.

**Figure 4:**
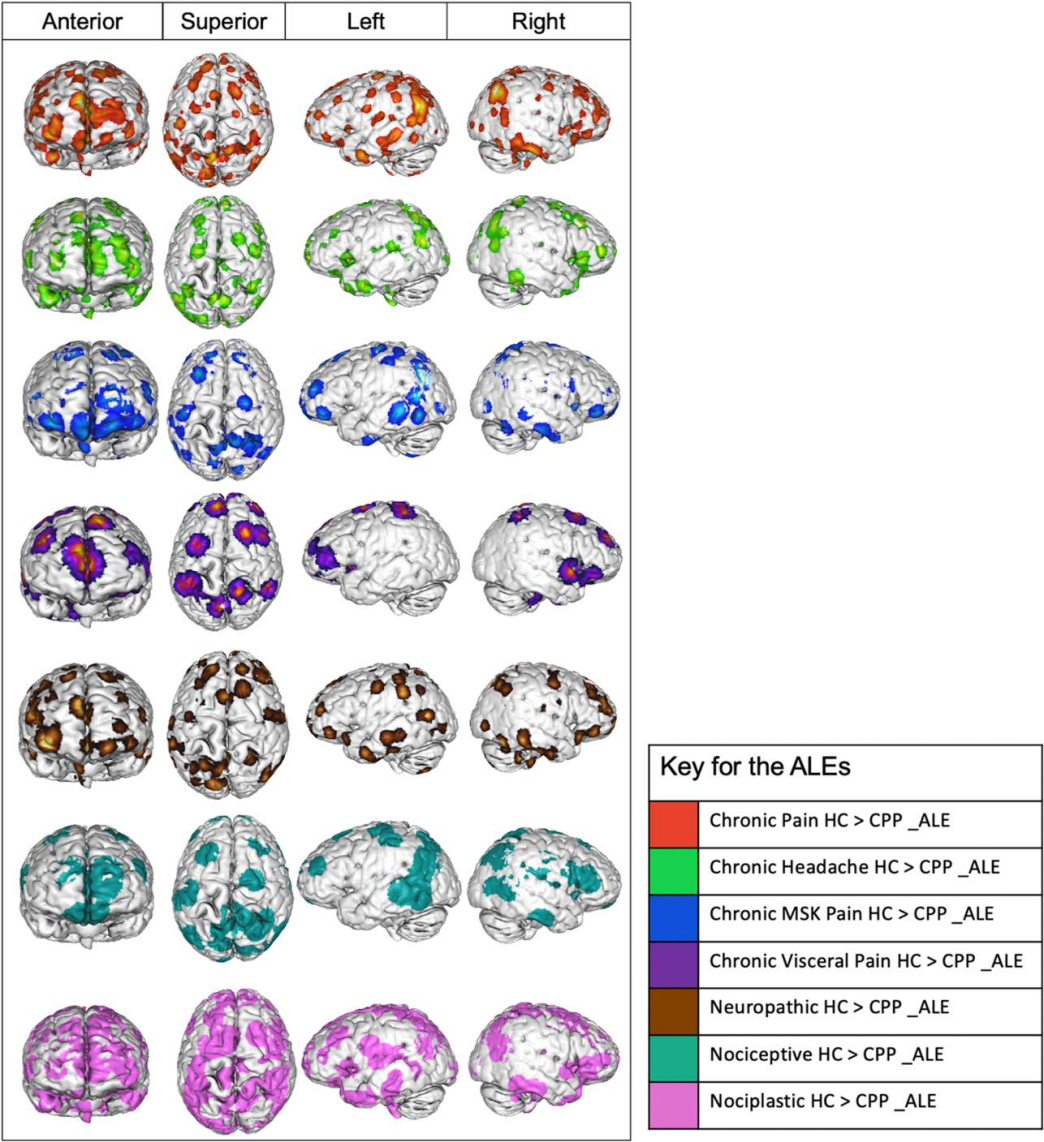
**ALEs overlapped on 3D brain images.** Illustrations of the seven ALEs overlapped on three-dimensional normalized brain templates generated by Multi-image Analysis GUI (Mango) 4.0.1.^45,46^ Each ALE has been represented in four planes: anterior, superior, left, and right to provide clear distinctions in the anatomical coverage between the seven ALEs. HC: healthy controls, CPP: chronic pain patient, ALE: activation likelihood estimation.

**Figure 5:**
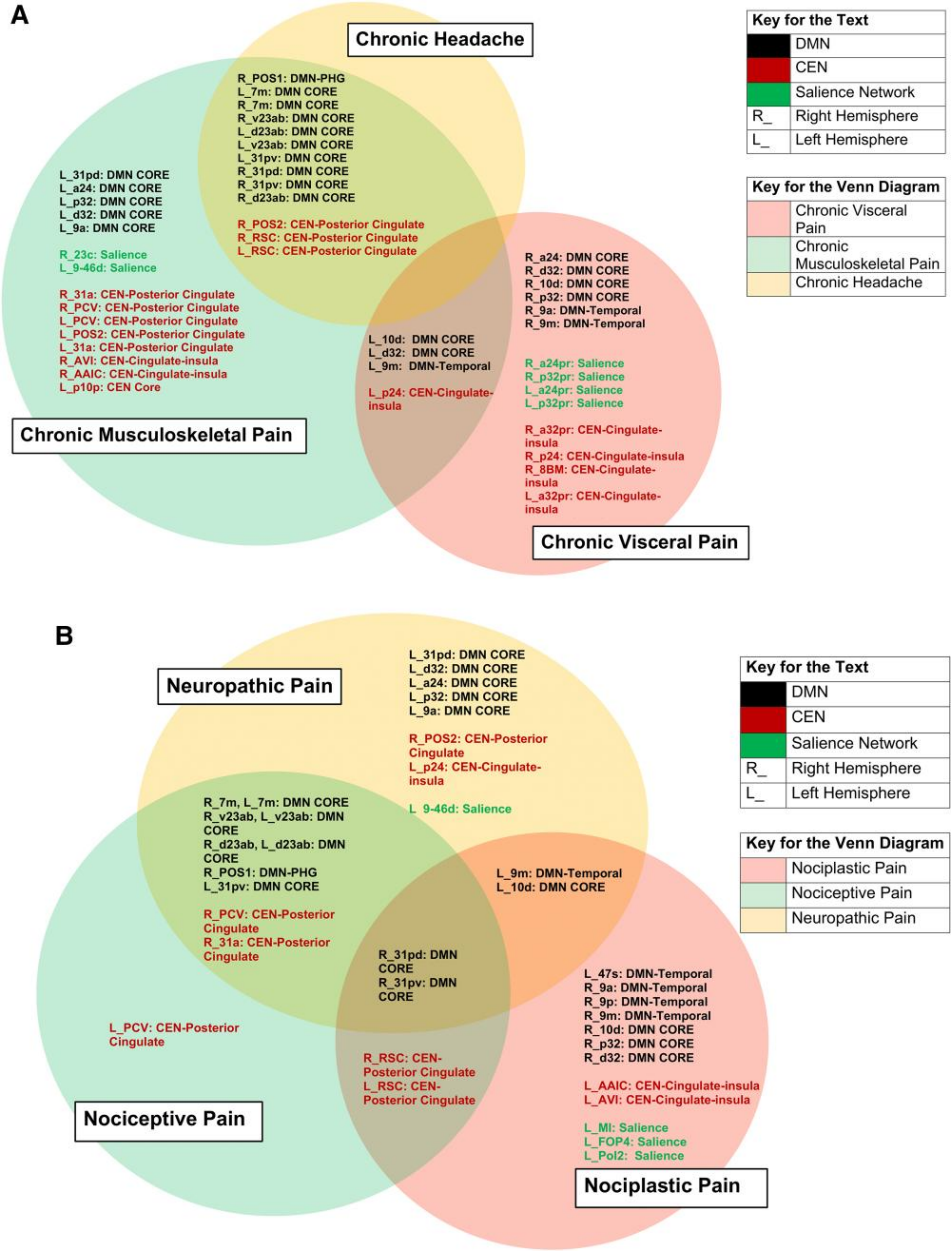
**Comparing chronic pain conditions and chronic pain mechanisms.** Venn diagrams reflecting (**A**) disease-specific network, subnetwork and parcellation overlap and non-overlap between chronic headache, chronic musculoskeletal and chronic visceral pain, and (**B**) Mechanism-Specific Network, Subnetwork and Parcellation overlap and non-overlap between Neuropathic, Nociceptive and Nociplastic Pain. DMN: Default Mode Network, CEN: Central Executive Network.

To explore deficits in the brain of a chronic pain patient within a global network framework, we assigned each parcellation to its corresponding core affiliate network based on Yeo’s 7-network model of the human cerebral cortex.^58^ Graph theory methods classify the brain as a complex meta-network, but to maintain clarity, we focused on seven major networks to discuss the chronic pain dynome. Each parcellation was labelled as one of the following core networks: the default mode (DMN), central executive (CEN), salience (SN), dorsal attention, limbic, sensorimotor, or visual networks.^58^ Subnetworks within these major networks were also constructed to provide more granular insights. We then analysed the network-based categorization of the parcellations and noted any salient asymmetries between chronic pain patients and healthy controls. Figures 3–5 summarizes the key methodological components involved in the study.

## Results

### Cluster-level analysis

A total of 61 studies met the inclusion criteria to be utilized in the meta-analysis,^19-23,51,59-113^ including a total of 2004 chronic pain patients and 1749 healthy controls. Supplementary Table 2 provides the chronic pain conditions in the included studies. More information about included studies, including patient gender, and their MNI coordinates are available in Supplementary Table 3.

Following cluster-level analysis, no significant clusters were detected at the thresholding value of 0.05 for the subcategory of chronic orofacial pain (HC > CPP), resulting in its exclusion from the final analysis. Table 3 demonstrates the seven statistically significant ALEs that were generated across studies, with their corresponding participant and experiment numbers. Figure 4 provides anatomical surface representations of the 7 ALEs. Detailed information about the ALEs, their contributing clusters, centred MNI coordinates, cluster volumes, and associated foci and gyri is provided in Supplementary Table 4 and Supplementary Document 1. Supplementary Fig. 1A–G provides two-dimensionsional illustrations of the underactive parcellations for all 7 ALEs.

**Table 3:** Key statistical figures for the seven statistically significant ALEs

		Subcategories	Contrast	Generated ALE Name	Participant number	Experiment number
Primary Meta-analysis	Chronic Pain	Chronic Pain	HC > CPP	Chronic Pain HC > CPP _ALE	CPPn = 2004 HCn = 1749	*n* = 61

Secondary Sub-analyses	Chronic Pain Mechanisms	Neuropathic	HC > CPP	Neuropathic HC > CPP _ALE	CPPn = 521 HCn = 448	*n* = 18
		Nociceptive	HC > CPP	Nociceptive HC > CPP _ALE	CPPn = 554 HCn = 456	*n* = 12
		Nociplastic	HC > CPP	Nociplastic HC > CPP _ALE	CPPn = 902 HCn = 793	*n* = 30

	Chronic Pain Conditions (ICD-11)	Chronic MSK Pain	HC > CPP	Chronic MSK Pain HC > CPP _ALE	CPPn = 765 HCn = 553	*n* = 16
		Chronic Headache	HC > CPP	Chronic Headache HC > CPP _ALE	CPPn = 461 HCn = 394	*n* = 17
		Chronic Orofacial Pain	HC > CPP	No ALE	nil.	nil.
		Chronic Visceral Pain	HC > CPP	Chronic Visceral Pain HC > CPP _ALE	CPPn = 219 HCn = 175	*n* = 8

ICD-11, International Classification of Diseases-11; CPPn, Chronic Pain Patient number; HCn, Healthy Control number Participant number: No of participants contributing to the ALE, Experiment number: No of experiments contributing to the ALE.

Statistical significance is determined by ALE.

### Chronic pain is a dysfunction of the DMN

The principal assessment involved using a meta-analysis to identify convergent differences in spontaneous resting-state brain activity between chronic pain patients and healthy controls. All studies meeting the inclusion criteria were combined irrespective of pain condition to identify the core anatomy of the chronic pain dynome. At our predefined statistical threshold (voxel height, *P* < 0.05 and familywise error–corrected cluster significance, *P* < 0.05), we identified that the majority of the disruptions largely involved the DMN (79.3%), followed by the CEN (20.7%).

To understand the anatomical components of these networks specifically implicated, we further magnified the networks based on the stratification of seventeen previously reported networks.^58^ Specifically, DMN involvement can be broken down into three subdivisions: Core DMN (78.3%, a24, p32, 10d, d32, s32, 31pv, 31pd, 7m, v23ab, d23ab), Temporal DMN (13.0%, 9m, 9a), and Parahippocampal DMN (8.70%, POS1). The CEN cortical involvement was distributed over two subnetworks: CEN-Posterior Cingulate (60%, POS2, RSC[retrosplenial cortex], PCV[precuneus visual-related subdivision]), and CEN-Cingulate Insula/Anterior-Cingulate (40%, p24).

These results were also translated to an alternate anatomical map: the cortical lobar and sub-lobar distribution.^114^ Three clusters contributed to the Chronic Pain ALE (HC > CPP). Cluster 1 showed involvement from the Medial Frontal Gyrus (52.7%), Anterior Cingulate (24.9%), Superior Frontal Gyrus (19.8%), and Middle Frontal Gyrus (2.5%). Cluster 2 involved the Lentiform Nucleus (51.6%), Caudate (22.9%), Insula (6.4%), Claustrum (6.2%), Anterior Cingulate (5.1%), Inferior Frontal Gyrus (2%), and Medial Frontal Gyrus (1%). Cluster 3 covered the Posterior Cingulate (43.9%), Precuneus (36%) and Cingulate Gyrus (20.2%) (Supplementary Table 4). This cluster analysis shows that chronic pain predominantly involves disrupted activity in the fronto-limbic lobes of the cortex, with minor contribution from the parietal and occipital lobes, and sub-lobar structures.

A post-hoc analysis was conducted using a more stringent statistical threshold (voxel height, *P* < 0.001 and familywise error–corrected cluster significance, *P* < 0.05) to confirm the reliability of our findings and the specificity of the cortical regions implicated above. We found our results to be consistent, with the Core DMN contributing to the majority of disrupted regions (Supplementary Table 5).

### Disease-specific arrangement of cortical regions within the chronic pain dynome

Beyond the primary chronic pain dynome, we explored the possibility that different chronic pain conditions could have different anatomical configurations within the dynome, given the heterogeneity of this disease process. We approached this by assessing disease-specific networks and parcellations, identifying overlaps in key cortical areas of interest. The ICD-11 classification system provided a pragmatic solution to the myriad of chronic pain conditions in the included studies. Figure 5A provides a concise summary of all the statistically significant findings obtained for the three chronic pain conditions, viewed through three progressively granular lenses: major networks, subnetworks and parcellations.

Chronic headache is primarily involved in disruptions in the DMN and the CEN, with the DMN largely centered on the Core DMN, and the CEN restricted to the posterior cingulate regions. Similarly, chronic MSK pain involved the Core DMN and CEN-Posterior Cingulate, while also extending into the SN regions. Finally, visceral pain was localized to the Core DMN and SN and CEN. Unlike chronic MSK pain, visceral pain uniquely involved the CEN-cingulate insula and anterior cingulate regions. These findings highlight a notable distinction: chronic visceral pain exhibits distinct cortical changes compared to chronic headache and MSK pain.

### Mechanism-specific arrangement of cortical regions within the chronic pain dynome

Similar to the disease-specific configuration, we also observed mechanism-specific alignments of the chronic pain dynome. We classified the mechanism of chronic pain into three groups: (i) nociceptive, (ii) neuropathic and (iii) nociplastic. While nociceptive and neuropathic pain are well-established, the concept of nociplastic pain has gained increasing attention.^47^ Nociplastic pain involves altered nociception in the absence of tissue damage or identifiable pathology in the somatosensory nervous system and is most often associated with secondary pain disorders. Our study sought to identify potential variations in network and parcellation distribution across these specific chronic pain mechanisms. Figure 5B succinctly summarizes the obtained results.

The DMN and CEN networks are consistently involved across all three pain mechanisms. However, a more detailed analysis reveals a key observation: the degree of overlap and non-overlap in anatomical parcellations between the mechanisms, as shown in Fig. 5B. Both neuropathic and nociplastic pain exhibit a mix of overlapping and distinct regions, whereas nociceptive pain is primarily characterized by overlapping regions. These findings suggest that neuropathic and nociplastic pain are associated with distinct cortical changes, while nociceptive pain is less likely to have a unique cortical distribution.

### Risk of bias assessment and sensitivity analysis

The data in this meta-analysis showed minimal heterogeneity, indicating that effect sizes remained consistent even after the sequential exclusion of studies, confirming the robustness of the findings. The overall risk of bias was low for most studies, though a few exhibited critical biases related to confounding factors and selective reporting of results. Details on the risk of bias are presented in Supplementary Fig. 2.

## Discussion

This report details the first ALE CBMA of 61 resting-state studies that helped generate an anatomically concise multinetwork dynome of chronic pain. We attempted to assess chronic pain patients’ undisturbed intrinsic state of pain, instead of predicting responses to stimuli that invoked pain. Ultimately, we found that pain syndromes largely involve network dysfunction in the DMN, more specifically, parcellations localized to the core DMN, suggesting that chronic pain largely reflects abnormal cognitive control on the internal processing of pain.^115^ To explore the heterogeneity of chronic pain, we characterized the chronic pain dynome through two different perspectives: (i) the ICD-11 disease sub-classification^5^ (chronic headache, MSK and visceral pain) and (ii) the specific pain mechanisms (nociceptive, neuropathic and nociplastic). While pronounced disparities were evident in the networks and parcellations implicated by both perspectives, our results suggest an overarching tripartite network dysfunction in three large-scale cognitive networks: DMN, CEN, and the SN. Dysfunction in these cognitive control networks likely illustrates an inappropriate allocation of cognitive resources that emphasizes focus on internal, passive sensory processing, which similarly reflects the psychopathological mechanisms in a variety of psychiatric and neurological disorders.^116^ This mirrors the emerging idea that some forms of chronic pain can be effectively treated through psychological mechanisms.^117^

The HCP parcellation scheme has specific advantages, such as improved spatial resolution and more accurate anatomical localization, which enhance the robustness of our findings. These advantages include a finer granularity in distinguishing brain regions and a higher level of detail in mapping functional connectivity, which are critical for identifying subtle differences in network dynamics associated with chronic pain. However, we also acknowledge that the described networks are well known and could be detected with other atlases/parcellation schemes.

### The DMN is the chronic pain centre

We observed that the DMN was the fundamental hub of the chronic pain dynome. The DMN is a large-scale brain network consisting of multi-modal association cortices which integrate information from brain regions for passive sensory processing.^118^ Generally, it is described as the ‘internal mind’ functioning at rest, thus not actively involved in attentional processing or goal-oriented tasks.^118,119^ Since we reported resting-state signal alterations, our results potentially demonstrate that chronic pain patients show intrinsically reduced activity in the DMN during episodes of spontaneous pain. Our results support several key functional neuroimaging studies, which have suggested that a constant state of chronic pain results in maladaptive plasticity of the DMN dynamics.^10,11,120-122^ Based on our findings, we would expect a person at rest to have a hyperactive DMN as theoretically they are in a state of passive thinking.^118,119^ However, for chronic patients, an underactive DMN reflects an entirely opposite state of abnormal cognitive alertness. This could explain sensations of spontaneous pain and pain hyperawareness, which can be thought of as active processes requiring cognitive attention.^123^ Hyperawareness could reflect the central sensitization and wind-up phenomena observed in several chronic pain states, which are thought to reflect sensitized wide-dynamic range neurons, aberrant neuronal plasticity, and expansion of nociceptive fields.^124^

In addition to the DMN, we also observed relative underactivity of the CEN, the ‘external’ mind involved in goal-directed tasks and decision making.^125^ In healthy individuals, the ‘external’ CEN functions anticorrelated with the ‘internal’ DMN,^126,127^ therefore, an underactive resting-state CEN is expected, in contrast to the ‘high functioning’ DMN counterpart.^128^ Aberrations in this relationship have been noted in neuropsychiatric conditions,^129,130^ and internetwork-CEN underactivity has been documented in chronic pain literature.^131^ Aberrant Central Executive Network (CEN) and Cingulo-Opercular (or Cingulo-Marginal) Network underactivity could also account for the observed decline in cognitive function in chronic pain disorders.^132^

### Differentiation of chronic visceral pain from chronic musculoskeletal pain and chronic headache

The present network-based approach allowed us to conclude that chronic MSK pain and chronic headache include similar network dysfunction, whereas chronic visceral pain involves specific differences. First, Chronic MSK pain and Chronic Headache involve underactive CEN-Posterior Cingulate Regions, whereas Chronic Visceral Pain involves underactive CEN-Cingulate Insula/Anterior-Cingulate Regions. Also, a core SN cluster stands out in chronic visceral pain compared to the non-core regions in chronic MSK pain (Fig. 5A).

Recently, Rebollo *et al*. mapped out a cortical network synchronized to the gastric rhythm by coupling electrogastrogram signals and fMRI BOLD fluctuations. Their pivotal finding was that signals corresponding to visceral sensations were specifically localized in the underlying operculum covering the insula.^133^ These results are concordant with both of our findings, as the operculum is a core area of the SN,^115^ and the insular involvement supports the CEN-Cingulate Insula underactivity we observed in our analysis.

Both Chronic Headache and Musculoskeletal Pain exhibited substantial overlap in their anatomical cortical distribution and in their subnetwork involvement: CEN-Posterior cingulate and core DMN. Based on the CEN, SN, and core DMN distributions, it appears that visceral pain exclusively involves Cingulate-Insula/Anterior-Cingulate regions, whereas chronic headache and MSK pain involve the posterior cingulate cortex. According to Bromm’s principles,^134^ such anatomical distinctions may suggest that visceral pain has a stronger emotional aspect, while musculoskeletal and chronic headache have a greater primary sensory aspect. It also supports the association of chronic visceral pain with mood disorders such as anxiety and depression.^135^

## Strengths and limitations

There were several strengths within this study. We used a methodical approach to finding published studies to increase the likelihood of identifying all relevant chronic pain studies. Bias in this review process was reduced through the implementation of a protocol requiring independent completion of screening, risk of bias assessments, and data extraction by two or more distinct reviewers. Additionally, the use of ALEs provides a more rigorous quantitative result than current diffusion tractography, which strengthens our findings.^136,137^ While a certain amount of individual variability is inevitable, an ALE-based approach allows for a qualitative demonstration of major white matter connectivity between regions.^138^

A more comprehensive meta-analysis could have been undertaken to encompass studies with multiple follow-ups and demographic variables, such as gender, and to address the correlation between these variables. The inclusion of demographic information is particularly important, as male and female brains demonstrate varied network responses in the context of chronic pain, an important difference that lends itself to a comprehensive analysis. Integrating this information into our meta-analysis would increase its thoroughness. As it stands, our study’s goal was to elucidate general trends that are likely representative of the broader population.

Further, we recognize that assessing chronic pain is complicated by its variable definition across the literature. Finally, although we acknowledge the importance of pain scale variability, our study's capacity to comprehensively address this aspect is limited.

## Conclusion

Taken together, our model of the chronic pain dynome is a prototype that can serve as a starting point for future research. Chronic pain involves complicated pathology, and studying its ‘dynomics’ will allow us to better understand how macroscale brain connections change in response to chronic pain.^18^ Using the HCP parcellation scheme, we were able to increase anatomic specificity. This allows for precise localization of brain regions associated with chronic pain. The detailed anatomical information provided by the HCP scheme further enables more accurate comparisons between studies, facilitating hypothesis refinement and advancing our understanding of chronic pain neuroanatomy. Our model of the dynome carries major clinical implications as functional connectomics are being successfully used to optimize brain function in neurological conditions.^139-142^ In the era of targeted neuromodulatory treatments, our results serve as an empiric basis for future investigation,^143,144^ with existing research already proving successful in the treatment of neuropathic pain, migraine, and fibromyalgia.^144,145^ To make our results clinically actionable, they would need to be incorporated into algorithms to identify patient-centric parcellation or network differences. This approach is still in its infancy, but our ultimate goal is to optimize existing successes in neuromodulatory treatments^146^ by improving their anatomical and target specificity for patient-centred care.

## Supplementary Material

fcaf343_Supplementary_Data

## Data Availability

Data used in this study are summarized in the supplementary information and are freely available in anonymized form in the referenced studies. Code used for analysis is available as Supplementary Document 2.
