# Supplementary material for: Multi-network dynamical structure of the human brain in the setting of chronic pain: a coordinate-based meta-analysis
Source: Brain Commun. 2025 Oct 29;7(5):fcaf343. doi: 10.1093/braincomms/fcaf343 (PMC12569763; doi:10.1093/braincomms/fcaf343)
Supplement: fcaf343_Supplementary_Data [file fcaf343_supplementary_data.docx]

##

## **Supplementary Files**

## **Supplementary Table 1**

| **Supplementary Table 1: Reasons for Article Exclusion in Title and Abstract Screening** | |
| --- | --- |
| **Reason for Article Exclusion** | **Number of Articles Excluded** |
| Conference Abstract | n = 415 |
| Therapy-Based Study | n = 689 |
| Review/Editorials/Comments/Book Chapter Series | n = 1117 |
| Irrelevant Study Objective | n = 685 |
| Acute Pain or Experimental Pain | n = 473 |
| Animal Study | n = 261 |
| Non-English Study | n = 2 |
| Non-Adult cohort | n = 100 |
| Non-fMRI study (e.g., PET, EEG) | n = 119 |
| Studies assessing Structural Changes | n = 147 |
| Specific Demographic (e.g., Veterans in Chronic Pain) | n = 49 |
| **Total** | **n = 4057** |
| *fMRI: functional Magnetic Resonance Imaging, PET: Positron Emission Tomography, EEG: Electroencephalography* | |

## **Supplementary Table 2**

| **Supplementary Table 2: List of Pain Conditions in the Included Studies** | |
| --- | --- |
| **Type of Pain Condition/ Clinical Diagnosis** | **Number of Studies with the Pain Condition** |
| Temporomandibular Disorder | n = 2 |
| Migraine | n = 12 |
| Osteoarthritis | n = 3 |
| Post-herpetic neuralgia/Herpes Zoster | n = 7 |
| Post-Spinal Cord Injury Neuropathic Pain | n = 1 |
| Chronic Neck and Shoulder Pain | n = 6 |
| Trigeminal Neuralgia | n = 5 |
| Fibromyalgia | n = 2 |
| Neuropathic pain | n = 1 |
| Chronic Back Pain/Leg Pain | n = 6 |
| Post-Cancer pain | n = 1 |
| Carpal Tunnel Syndrome | n = 1 |
| Inflammatory Bowel Disease | n = 5 |
| Cluster Headache | n = 2 |
| Medication Overuse Headache | n = 1 |
| Primary Dysmenorrhoea | n = 2 |
| Tension Type Headache | n = 1 |
| Chronic Pelvic Pain | n = 1 |
| Non-specific Chronic Pain | n = 2 |
| **Total** | **n = 61** |

| **Supplementary Table 3:** | **Article** | **Pain Condition**  **Detailed table summarizing data from the 61 studies that met inclusion criteria to be used in the meta-analysis** | | **Number of Patients/ Controls** | **Number of Patients/ Controls: Gender (M/F)** | **Experiment Analysis Methodology** | **Pain Duration** | **Pain Duration Statistic** | **Medications** | **Original Coordinates** |
| --- | --- | --- | --- | --- | --- | --- | --- | --- | --- | --- |
|  | Lim, et al. 2021^1^ | Chronic Temporomandibular Disorder (TMD) | | 12/24 | (1M/11F)/(5M/9F) | ALFF | 8 years (min = 1.5 years, max = 22 years) | Mean (min/max) | Participants took stable doses of pain medications for at least 4 weeks and had to be willing to limit introduction of new medications for TMD management during the study. | 14 48 34  42 30 22  42 32 22  42 30 20  50 -10 34  44 -24 48  12 50 32  42 30 22  38 -30 58  38 18 28  50 -12 34  44 -24 48 |
|  | Lim, et al. 2021^2^ | Chronic Migraine | | 20/26 | (6M/14F)/ (7M/19F) | BOLD Signal Variability (BOLDsv) | 12.7 ± 1.6  Years | Mean ± SD | Patients were included only if they reported no opioid or hormonal contraceptive use 6 months before enrolment. | 44 22 36  38 -54 44  38 -54 44  44 22 36  40 -54 44  -50 -62 20  44 24 40  38 -56 44  50 -60 16  -40 -56 46 |
|  | Barroso, et al. 2021^3^ | Chronic Osteoarthritis | | 46/36 | (16M/30F)/ (8M/ 38F) | Hub Topology | 6.8 ± 5.45 Years | Mean ± SD | No Data | -2 35 31  52 -59 36  36 10 1  59 -17 29  37 1 -4 |
|  | Huang, et al. 2020^4^ | Postherpetic Neuralgia  and Herpes Zoster and | | 52/20 | (27M/25F)/ (7M/13F) | ALFF | 227.0 ± 72.1 Days | Mean ± SD | Baseline treatment of 8mg amitriptyline and 75mg pregabalin | 9 -57 24  -3 -63 27  -6 -48 27  66 -6 -27  69 -36 -12  54 -51 36  9 -48 24  -6 -48 27  0 -63 27  42 48 27  63 -6 -27 |
|  | Park, et al. 2020^5^ | Neuropathic pain | | 41/33 | (28M/13F)/-- | fALFF | 408.8 ± 323.9 days | Mean ± SD | See Table S1 in Supplementary file attached to published paper “Alterations in power spectral density in motor- and pain-related networks on neuropathic pain after spinal cord injury” for exhaustive list of medications for every participant. | -4 34 38  38 34 46  -2 12 64  -2 50 24  5 34 -4  4 48 -6  -4 34 38 |
|  | Liu, et al. 2020^6^ | Ankylosing Spondylitis (Chronic Back Pain) | | 54/53 | (39M/15F)/ (38M/12F) | -Resting State Functional Connectivity  -Degree Centrality  -Betweenness Centrality | 7.88 ± 6.89 Years | Mean ± SD | 38 Patients with chronic pain were taking nonsteroidal anti-inflammatory medications (Meloxicam, Ibuprofen, Diclofenac and Celebrex). Patients had not taken any other biologic agents or central analgesics in 2 months prior to study. | 19 -8 64  8 -48 31  -3 44 -9  -22 7 -5  23 10 1  17 -28 -17  10 -46 73  19 -8 64  -7 51 -1 |
|  | Yue, et al. 2020^7^ | Chronic neck and shoulder pain | | 28/25 | (17M/11F)/(13M/12F) | ALFF | 32.56 ± 27.72  Months | Mean ± SD | Patients did not receive any pain treatment prior to commencement of the study. | -51 -33 0  -9 -66 27  -42 -18 60  9 -36 24  33 -72 33  24 21 36  30 -45 60 |
|  | Dai, et al. 2020^8^ | Postherpetic neuralgia | | 12/12 | (7M/5F)/(7M/5F) | ALFF + fALFF | No info. | No info. | No info. | 9 -30 -45  42 0 9  -48 -21 -3  -12 45 -6  21 -45 18  12 15 33  -12 -57 51  -15 45 -6  15 18 60 |
|  | Li, et al. 2020^9^ | Chronic shoulder pain | | 37/24 | (20M/17F)/(10M/14F) | ReHo | 6.38±6.05  Months | Mean ± SD | Patients were excluded if their pain was therapeutically treated with analgesics. | 6 60 -12 |
|  | Zhang, et al. 2019^10^ | Trigeminal neuralgia | | 29/34 | (10M/19F)/(13M/21F) | ALFF | 6.02 ± 4.35 Years | Mean ± SD | Patients were instructed to cease medications 24h before commencement of study | -57 0 -24  -54 -60 12  0 60 -3  -6 -57 66  42 -3 63 |
|  | Yan, et al. 2019^11^ | Trigeminal Neuralgia | | 28/28 | (14M/14F)/(14M/14F) | dynamic regional homogeneity (dReHo) | 4.5 ± 13.3 Years | Mean ± SD | Patients were excluded if they were on any psychiatric medications | -42 -60 9  -15 -72 51  -39 -6 66 |
|  | Zhang, et al. 2019^12^ | Chronic low back-related leg pain | | 25/27 | (10M/15F)/(15M/12F) | Voxel-mirrored homotopic connectivity | 38.96 Months | Mean | Included patients who failed to respond to conservative medications such as anti-inflammatory drugs and acetaminophen without trialling opioids, physical therapy, and exercise. | 54 -15 -36  9 12 -9  30 54 -3  55 -24 0  18 57 9  15 21 -18  48 36 -6  60 -33 -6  3 -57 27  60 -6 -33  15 6 -6  9 12 -9  42 -75 0  12 15 -6  18 51 10  3 -48 51  33 -12 51  18 18 0  42 45 18  15 42 51  -54 -15 -36  -9 12 -9  -30 54 -3  -55 -24 0  -18 57 9  -15 21 -18  -48 36 -6  -60 -33 -6  -3 -57 27  -60 -6 -33  -15 6 -6  -9 12 -9  -42 -75 0  -12 15 -6  -18 51 10  -3 -48 51  -33 -12 51  -18 18 0  -42 45 18  -15 42 51 |
|  | Kaplan, et al. 2019^13^ | Fibromyalgia | | 40/46 | (--/40F)/(--/46F) | Eigenvector Centrality | No data | No data | See Supplementary Table 4 attached to paper **“**Functional and neurochemical disruptions of brain hub topology in chronic pain” for exhaustive list of medications.  *9 patients missing medication data | 65 -24 -19  -58 -26 -15  6 54 16  -58 -30 -4 |
|  | Gu, et al. 2018^14^ | Postherpetic neuralgia | | 18/18 | (11F/7M)/(11F/7M) | ALFF | 116.78 ± 25.96  days | Mean | No info | 30 57 0  42 33 33  3 93 9  -27 9 69  -27 57 57  -12 -75 -15  3 -93 9  6 66 27 |
|  | Rogachov, et al. 2018^15^ | Chronic back pain | | 71/62 | (51M/20F)/(43M/19F) | ALFF | No info | No info | 42 patients were treated with biological agents/anti-TNF-α medication (Simponi, Remicade, Embrel and Humira), and 27 with nonsteroidal anti-inflammatory medication (Naprosyn, Voltaren and Celebrex). | 16 -26 16  -14 -28 16  18 -24 14 |
|  | Yuan, et al. 2018^16^ | Trigeminal Neuralgia | | 23/23 | (14M/9F)/(12M/11F) | fALFF  ReHo | 5.69 ± 3.33 Years | Mean | Patients did not receive any preventative treatments prior to the study. | 9 -48 0  -42 -12 6  -21 -72 -6  33 -54 -30 |
|  | Hong, et al. 2018^17^ | Postherpetic neuralgia | | 55/28 | (22M/33F)/(9M/19F) | Functional connectivity density (FCD) | 11.24 ± 6.67 Days | Mean ± SD | Chronic pain patients were instructed not to take antidepressants of antipsychotics before the scans. | -6 -75 51  -9 -75 48 |
|  | Cao, et al. 2017^18^ | Postherpetic neuralgia | | 73/55 | (39M/34F)/(31M/24F) | fALFF  + ReHo | 12.2 ± 3.7 Months | mean ± SEM  SEM: standard error of mean. | Chronic pain patients were instructed not to take antidepressants of antipsychotics before the scans. | 21 -84 -33  51 -30 -24  -60 -36 33  0 45 -6  48 21 -12  18 -102 3  -6 -99 6  -45 -63 -3  39 -69 48  36 -78 -45  69 -33 -21  27 -48 15  48 21 -12  3 66 21  -24 -51 15  -63 -36 36  51 -63 42  -24 -48 18  48 -39 -33  -57 -33 48  0 -9 48  24 -48 12  3 -81 -6  -21 -60 6  -45 18 -12  3 57 15  3 -18 27  15 27 66 |
|  | Cao, et al. 2017^19^ | Postherpetic Neuralgia | | 19/19 | (11M/8F)/(8M/11F) | ReHo  +  FALFF | 5.4 ± 1.3  Years | mean ± SEM  SEM: standard error of mean | Nil. | -36 -60 -45  51 -33 -30  54 -3 24  -39 -42 -9  -21 -36 -9  -63 -21 -9  -51 -21 21  57 -15 18  54 -6 42  -36 -57 60  36 54 15  -30 -69 42  30 36 39  -24 36 54 |
|  | Yu, et al. 2017^20^ | Chronic neck and shoulder pain | | 25/20 | (13M/12F)/(10M/10F) | ReHo | No info | No info | No treatment was administered that could impact neurological function. | -39 -5 7  21 -47 61  20 -44 61  -13 21 48  -12 -9 53  -2 3 37 |
|  | Wang, et al. 2015^21^ | Trigeminal neuralgia | | 17/19 | (7M/10F)/(9M/10F) | ReHo | 6.98 ± 5.64 Years | Median ± SD | No info. | 51 -57 -15  -12 3 -30  -9 -87 -21  12 -12 -21  6 -15 6  39 -45 42  -21 -51 69 |
|  | He, et al. 2014^22^ | Temporomandibular Disorder | | 23/20 | (9M/14F)/(9M/11F) | fALFF | No info | No info | Patients did not take any medications before the scan. | -38 -19 62  -10 1 74  -4 10 57  -46 22 34  31 23 -26 |
|  | Zou, et al. 2021^23^ | Migraine | | 17/20 | (7M/10F)/(9M/11F) | RsFc | 10.47 ± 3.99 | Mean ± SD | Patients were excluded for overuse of pain medication | -24 30 36  6 -72 6  0 -57 15  -21 54 15 |
|  | Li, et al. 2020^24^ | Migraine | | 72/46 | (15M/57F)/(12M/34F) | fALFF | 61.67 (44.38, 78.95) | mean (95 %CI) | No prophylactic headache medication or acupuncture treatment 3 months prior to study | -5 -24 -51  9 -6 6  -27 0 -15  54 -66 33 |
|  | Lan, et al. 2020^25^ | Knee Osteoarthritis | | 23/23 | (8M/15F)/(9M/14F) | ALFF | No info | No info | No info | -11 -66 51  12 -76 47  -54 -60 30  53 -54 38  -12 63 12  13 59 20 |
|  | Zhao, et al. 2017^26^ | Somatoform Pain Disorder | | 13/23 | (5M/8F) (12M/11F) | RsFc | 3.5 ± 2.57  Years | Mean ± SD | Patients were requested to stop their pain medications 1–3 days before fMRI | 57 -48 15  12 48 18 |
|  | Wang, et al. 2017^27^ | Trigeminal neuralgia | | 17/19 | (7M/10F)/(8M/11F) | ALFF | 6.98 ± 5.64 Years | Mean ± SD | All patients were given carbamazepine 300–900 mg daily | 60 -21 -27  -6 45 24  -60 -27 -24  60 -24 -27  -6 45 24  24 66 3  15 -84 -6  -9 45 15 |
|  | Huang, et al. 2016^28^ | Somatoform Pain Disorder | | 13/23 | (5M/8F)/ (12M/11F) | ReHo | See Table 1 for exhaustive list for patients. | See Table 1 for exhaustive list for patients. | No electroconvulsive therapy 4 weeks prior to study commencement. | 48 -21 36  -48 -24 36  1 -84 1  -6 -87 9  6 -78 -36 |
|  | Pujol, et al. 2014^29^ | Fibromyalgia | | 40/36 | (--/40F)/(4M/36F) | Connectivity Degree Mapping | 7.2 ± 4.7 Years | Mean ± SD | Patients allowed to continue stable current medical treatment, but advised not to take rescue analgesic drugs (e.g. nonsteroidal anti-inflammatory drugs and Panadol), 72 hours prior to fMRI | -8 -30 70  -59 -8 23  -59 -20 16  -46 -38 22  -56 -15 8  -8 -91 30  -8 -53 -4  -46 -76 30  48 -61 30  -8 46 36  4 -15 16  -21 0 -4  -27 -15 -25 |
|  | Ihara, et al. 2019^30^ | Chronic Neck Pain | | 20/20 | (6M/14F)/(6M/14F) | RsFc | 47.2 ± 40.7  Months | Mean ± SD | Study did not control for medications such as: Tramadol, pregabalin or antidepressants | 2 -36 36  60 -19 -22  3 -59 33  -1 -46 30  0 -47 12  -51 -59 27  -14 59 20  -25 20 56  -1 -57 24  26 27 49  1 -38 77  -8 -53 60  2 -36 36  48 -59 41  -6 -95 19  4 -35 -15  52 7 20  23 -53 57 |
|  | Liao, et al, 2016^31^ | Cancer-induced Bone Pain | | 12/12 | N/A | ALFF | No info | No info | Patients using analgesics advised to cease medication 1 week prior to commencement of study. | 0 -84 -9  -6 -66 3  57 -72 9  54 -42 12  51 27 15 |
|  | Kolesar, et al. 2017^32^ | Failed Back Surgery Syndrome  Chronic Back Pain | | 11/11 | (6M/5F)/(6M/5F) | RsFc analysis | No info | No info | No info | 37 -59 38  -5 21 43  -52 -56 37  -29 -51 -46  6 67 -5 |
|  | Lu, et al. 2017^33^ | Carpal Tunnel Syndrome | | 28/24 | (2M/26F)/(3M/21F) | ALFF | 4.79 ± 1.28 Months | Mean | No info | -57 -12 39  51 -6 57 |
|  | Hodkinson, et al. 2016^34^ | Osteoarthritis | | 40/40 | (10M/30F)/(10M/30F) | ALFF and fALFF | 15 ± 9 Years | Mean ± SD | Patients on daily medication /prophylactic treatment were excluded | 6 62 -20  48 44 10  -28 28 34  20 6 22  16 -14 18  22 -20 22  6 -20 62  10 -38 56  2 -22 -30  -18 -36 -32  40 -18 12  34 -22 16  50 -22 20  -54 -46 2  -58 -32 14  4 -20 58 |
|  | Ma, et al. 2015^35^ | Irritable Bowel Syndrome | | 21/21 | (14M/7F)/(11M/10F) | ALFF | 4.92 ± 3.07  Years | Mean ± SD | Patients were excluded if they were taking centrally acting drugs such as pain medications, antidepressants, anxiolytics. | -30 3 66  63 9 -3  18 -9 -15  15 -45 75  42 15 57  -36 -36 69 |
|  | Li et al, 2017^36^ | Migraine | | 69/42 | (7M/62F)/(8M/34F) | ALFF | 62.91 (43.91; 81.84)  Months | Mean (95%CI) | Patients agreed that they would not take any medications for migraine treatment during the entirety of the study. In extreme pain, an exemption was made for ibuprofen as a rescue medication. | 30 -75 24  -27 -69 24  -3 -33 -57 |
|  | Chen, et al. 2018^37^ | Chronic Neck Pain | | 104/96 | (45M/59F)/(50M/46F) | ReHo | 37.49 ± 24.93  Months | Mean ± SD | Patients received acupuncture therapy for 1 month during the study. | 60 -48 6  -51 -27 42 |
|  | Zhang, et al. 2017^38^ | Migraine | | 29/31 | (8M/22F)/(9M/22F) | ALFF/ReHo | 9.6 ± 6.83  Years | Mean ± SD | Patients were excluded if they took preventative medication | 42 -3 60  45 -39 63  42 0 60  -27 -36 54  36 -42 63  39 0 63  -33 -45 60  21 -36 78 |
|  | Li, et al. 2017^39^ | Chronic Back Pain (Ankylosing Spondylitis) | | 27/28 | (18M/9F)/(17M/11F) | ALFF | No info | No info | No info | -6 66 -15  54 -12 9  18 -57 3 |
|  | Gao, et al. 2016^40^ | Migraine | | 55/44 | (--/55F)/(--/44F) | Functional Connectivity Density mapping | 10.2 ± 7.6  Years | Mean ± SD | Patients were excluded if they had taken prophylactic medications 3 months prior to the study, and had a record of long-term use of analgesics | -15 33 33  -33 3 -18  -39 21 3  -39 36 15  -27 36 -18  12 33 15  -33 15 3  39 -15 -3  -30 -33 0  39 -24 -9  -27 12 -3  24 15 3  -12 18 12  21 18 6  24 0 -18  60 -39 -15 |
|  | Ao, et al. 2021^41^ | Irritable Bowel Syndrome | | 13/14 | (8M/5F)/ (8M/6F) | fALFF | 16.6 ± 5.10  Months | Mean ± SD | Patients on regular analgesics were excluded. | -18 39 24 |
|  | Chou, et al. 2017^42^ | Cluster Headache | | 17/18 | (15M/2F)/(10M/8F) | RsFc | 12.1 ± 5.8  Years | Mean ± SD | Patients were instructed not to take medications 24hrs prior to the study. | -34 14 0  8 16 32 |
|  | Chen, et al. 2017^43^ | Medication Overuse Headache | | 37/31 | (7M/30F)/(12M/20F) | Functional Density analysis | 18.57 ± 9.08  Years | Mean ± SD | Patients were excluded if they took psychoactive or hormonal medications. | 21 -18 -18  0 27 21  15 15 -24 |
|  | Liu, et al. 2021^44^ | Migraine | | 37/15 | (6M/31F)/(2M/13F) | ReHo | 16.19 ± 12.81  Years | Mean ± SD | Patients were excluded if they had prophylactic medications in the month prior to the study, or psychoactive/vasoactive agents in the 3 months prior to the study. | 18 -60 -42  -18 -63 -42 |
|  | Zhang, et al. 2019^45^ | Primary Dysmenorrhea | | 20/14 | (--/20F)/(--14F) | ALFF | No info | No info | Patients had to refrain from medications in the month prior to study commencement. | 18 -30 -21  12 -36 -33  33 -9 -42  -21 -45 51 |
|  | Zhou, et al. 2018^46^ | Chronic Lower Back Pain | | 20/25 | (13M/12F)/(15M/12F) | ALFF | 37.08 ± 10.23  Months | Mean ± SD | Patients had to report nonsteroidal anti-inflammatory drugs usage and acetaminophen, but without the use of opioids. | 51 -54 -21  6 0 -3  -18 57 9  -42 -36 51  36 -57 48 |
|  | Zhao, et al. 2013^47^ | Migraine | | 19/20 | (--/19F)/(--/20F) | ReHo | 10.15 ± 7.01  Years | Mean ± SD | Patients were advised not to take prophylactic medications 1 month prior to the commencement of the study. | -4 -6 52  12 -4 51  12 -45 37  -47 -20 14  41 12 5  -34 27 2  41 26 -2  -21 -4 64  23 -16 58  -10 3 57  7 51 44  -4 2 54  21 10 66  -56 -4 -23  41 -63 28  -30 -90 0  35 -87 3  -21 -78 19  6 -85 34  -21 -78 19  -10 -75 -7  18 -69 -4  -48 -56 47  38 -56 42  -53 -17 14  52 -17 15  -30 -75 36 |
|  | Zhang, et al. 2016^48^ | Migraine | | 22/22 | (9M/13F)/(9M/13F) | RsFc | 47.3 ± 42.1  Months | Mean ± SD | Patients were excluded if they had a history of prophylactic/therapeutic medication/ acupuncture 3 months prior to study commencement. They were also excluded if they had a long-term history of analgesics use. | -9 49 35  7 57 25  -3 63 17  7 59 22  -45 -68 41  49 -63 41  -6 51 11  7 52 5  -7 -54 27  3 -54 27  -7 -53 25  -45 -59 30  56 -51 30  -10 -57 35  3 -54 31  -21 58 22  22 58 21  -40 28 -16  41 28 -17  -54 -56 24  34 20 -25  -49 14 -30  52 10 -37 |
|  | Li, et al. 2020^49^ | Migraine | | 72/46 | (15M/57F)/(12M/34F) | ReHo | 66.75 (32.19–101.31)  Months | Mean (95%CI) | Patients were excluded if they took prophylactic medications or any acupuncture in the 4 months prior to study commencement. | 33 -75 27  21 -72 36  -30 -81 21 |
|  | Zhao, et al. 2014^50^ | Migraine | | 19/20 | (--/19F)/(--/20F) | ReHo | 9.1 ± 2.6 Years | Mean ± SD | Patients were required to cease migraine prophylaxis drugs 6 weeks prior to commencement of study but were allowed to take pirprofen when required. | -27 67 13  -3 21 -12  4 17 -10  -10 -12 46  6 -11 33  -18 -79 43  26 -75 36  -12 9 -15  16 15 -13  -10 -32 -30  -10 -16 -4  -22 -65 -14  -22 -41 -35 |
|  | Wang, et al. 2014^51^ | Tension Type Headache | | 10/10 | (5M/5F)/(5M/5F) | ReHo | 5.1 ± 6.8  Years | Mean ± SD | Patients were excluded if they took analgesics or other drugs within 2 weeks of study commencement. | -3 9 -3  3 9 -3  -21 30 51  -18 18 69  -15 -51 24  15 -48 21  -21 6 6  24 0 6 |
|  | Wu, et al. 2016^52^ | Primary Dysmenorrhea | | 46/49 | (--/46F)/(--/49F) | ReHo | 9.22 ± 2.85 Years | Mean ± SD | Patients were excluded if they used oral contraceptives, hormonal supplements, centrally-acting medications (e.g., opioids), Chinese herbal medication 6 months prior to the study. | 6 62 22  0 66 12  -6 50 4  -42 -38 52 |
|  | Zhou, et al. 2019^53^ | Chronic Low Back Pain and Leg Pain | | 25/26 | N/A | ReHo | 36.72 ± 9.63  Months | Mean ± SD | Patients were included if they showed failure to respond to conservative treatment within around a month prior to commencement of study e.g., anti-inflammatory drugs and acetaminophen  without use of opioids. | 12 15 -15  -30 45 9  12 12 -12  -21 45 18  24 57 -3  -6 27 15  -12 12 -9  -18 54 9  -51 -51 36  9 -12 -33  -15 63 -6  51 -48 21  -3 -48 33  -54 -51 12  -30 27 57  12 12 -12  -21 57 9  -6 30 15  0 -51 33 |
|  | Chen, et al. 2021^54^ | Irritable Bowel Syndrome | | 36/36 | (16M/20F)/(10M/26F) | ReHo  ALFF | 19.11 Months | Mean | Patients were excluded if they took antimotility drugs or antidepressants 2 weeks prior to study commencement. | 42 -39 36  6 -21 -12  -3 -54 18  42 -42 48  -30 39 -6  15 -3 51 |
|  | Ke, et al. 2015^55^ | Irritable Bowel Syndrome | | 31/32 | (26M/6F)/(25M/7F) | ReHo | 32.7 ± 23.6  Months | Mean ± SD | Patients did not take any peripheral or centrally acting medications for disease management. | 3 12 33  -3 16 27  6 30 -12  -3 21 -15  -9 45 48  12 12 -9  21 15 57  33 45 30  54 30 -12  -30 45 18  -42 33 9  -6 -60 57 |
|  | Li, et al, 2021^56^ | Irritable Bowel Syndrome | | 15/26 | (11M/4F)/(19M/7F) | ReHo  ALFF | 4.57 ± 3.90  Years | Mean ± SD | Patients were excluded if they used biologics, glucocorticoids, or psychotropics 6 months prior to the study commencement. | -30 -27 -9 |
|  | Jiang, et al. 2016^57^ | Postherpetic Neuralgia | | 18/18 | (11M/7F)/(11M/7F) | Voxel-mirrored homotopic connectivity | 116.78 ± 25.96  Days | Mean ± SD | No info. | 24 48 30  6 -51 42  -24 48 30  -6 -51 42 |
|  | Qui, et al. 2012^58^ | Cluster Headache | | 12/12 | (12M/--)/(12M/--) | ReHo | No info | No info | Patients were instructed not to take prophylactic medications | -43 24 28  53 11 48  57 -41 -26  25 68 9  -6 70 6  12 -54 27 |
|  | Chen, et al. 2019^59^ | Migraine | | 56/31 | (18M/38F)/(13M/18F) | ReHo | 7.41 ± 3.20  Years | Mean ± SD | Patients were excluded if they had history of overuse analgesics | -51 12 3  -33 57 3  51.07 23.98 3  -6 36 0  6 33 0  -25.21 8.48 0  27.8 4.6 0  6 -54 60  -3 -54 63 |
|  | Lin, et al. 2017^60^ | Chronic prostatitis/Chronic Pelvic Pain Syndrome | | 27/27 | (27M/--)/(27M/--) | ReHo | 4.0 ± 1.6 Years | Mean ± SD | Patients were excluded if they were treated for the symptoms | -6 27 24  9 15 33  -33 18 0  33 24 0  6 57 15 |
|  | Zhang, et al. 2021^61^ | Chronic Neck Pain | | 106/57 | (28M/78F)/(10M/47F) | ALFF | 84.96 ± 74.00 months | Mean ± SD | No info. | -60 -51 12  -60 -54 -6  -54 -66 27  -42 -63 54  -33 -66 39  -63 -36 0  -60 -54 -6  -57 -57 24 |
|  | | | ***Supplementary Table 3:*** *Detailed table summarizing data for the 61 studies that meet inclusion criteria to be used in the meta-analysis including: Article Name, Pain Condition, Number of Participants (Healthy Controls/Chronic Pain Patients), Experimental Analytical Strategy used by the respective study, Pain Duration of Participants, Pain Duration Statistic, Participant Medications and associated Instructions Regarding Medication Consumption, and Original MNI Coordinates used to generate the Activation Likelihood Estimations (ALEs).*  *ALFF: amplitude of low-frequency fluctuation, SD: Standard Deviation, fALFF: fractional amplitude of low-frequency fluctuations, ReHo: Regional Homogeneity, SEM: Standard Error of Mean, RsFc: Resting-state Functional-connectivity, fMRI: functional Magnetic Resonance Imaging.* | | | | | | | |

| **Sub-analysis** | **Subcategories** | **Contrast** **Supplementary Table 4:** | **Contributing**  **Foci** | **Clusters** | **Centred MNI Coordinates**  **Detailed data and statistical figures for the 7 sub-analyses** | **Volume** | **Gyrus** |
| --- | --- | --- | --- | --- | --- | --- | --- |
| **Chronic Pain** | Chronic Pain | HC > CPP | 488 | 3 | Cluster 1: -7.1, 52.9, 13.5  Cluster 2: 0.6, 13.1, -4.8  Cluster 3: 2.6,  -53.1, 26.7 | 16904 mm^^3^  14560 mm^^3^  10304 mm^^3^ | 52.7% Medial Frontal Gyrus, 24.9% Anterior Cingulate, 19.8% Superior Frontal Gyrus, 2.5% Middle Frontal Gyrus  51.6% Lentiform Nucleus, 22.9% Caudate, 6.4% Insula, 6.2% Claustrum, 5.1% Anterior Cingulate, 2% Inferior Frontal Gyrus, 1% Medial Frontal Gyrus  43.9% Posterior Cingulate, 36% Precuneus, 20.2% Cingulate Gyrus |
| **Mechanism** | Neuropathic | HC > CPP | 182 | 2 | Cluster 1: -0.4, -53, 34.4  Cluster 2: -12.9, 52.8, 15.5 | 8912 mm^^3^  8464 mm^^3^ | 60.6% Precuneus, 24.7% Posterior Cingulate, 14.6% Cingulate Gyrus  55.7% Medial Frontal Gyrus, 24.6% Superior Frontal Gyrus, 10.6% Anterior Cingulate, 8.7% Middle Frontal Gyrus |
|  | Nociceptive | HC > CPP | 72 | 1 | Cluster 1: 3.7, -46.4, 28.4 | 13968 mm^^3^ | 34.8% Precuneus, 27.4% Cingulate Gyrus, 20.1% Posterior Cingulate, 16% Thalamus |
|  | Nociplastic | HC > CPP | 235 | 3 | Cluster 1: -31.5, 15, -0.2  Cluster 2: 3.8, 56.7, 16.4  Cluster 3: 1.8, - 52.9, 25.7 | 7280 mm^^3^  6936 mm^^3^  5480 mm^^3^ | 66.9% Lentiform Nucleus, 13.2% Insula, 10.9% Claustrum, 5.9% Inferior Frontal Gyrus, 1.4% Parahippocampal Gyrus  66.7% Medial Frontal Gyrus, 26.8% Superior Frontal Gyrus, 6.5% Anterior Cingulate  53.3% Posterior Cingulate, 26.4% Precuneus, 20.3% Cingulate Gyrus |
| **ICD-11** | Chronic MSK Pain | HC > CPP | 142 | 3 | Cluster 1: 0, -51.4, 34.2  Cluster 2: 19.3, 11.6, - 5.7  Cluster 3: -16.3, 53.8, 8.9 | 10784 mm^^3^  8704 mm^^3^  7480 mm^^3^ | 53% Precuneus, 25.2% Cingulate Gyrus, 21.6% Posterior Cingulate  42.7% Lentiform Nucleus, 36.8% Caudate, 8.7% Claustrum, 4.4% Anterior Cingulate, 1.2% Subcallosal Gyrus  49.7% Anterior Cingulate, 27.1% Medial Frontal Gyrus, 18.1% Superior Frontal Gyrus, 5.1% Middle Frontal Gyrus |
|  | Chronic Headache | HC > CPP | 158 | 1 | Cluster 1: 2.8, -52.5, 27.5 | 6784 mm^^3^ | 41.4% Posterior Cingulate, 35.1% Precuneus, 23.5% Cingulate Gyrus |
|  | Chronic Visceral Pain | HC > CPP | 39 | 2 | Cluster 1: -7.5, 25.8, 26.5  Cluster 2: 1.6, 58.4, 13.3 | 14200 mm^^3^  8824 mm^^3^ | 68.8% Cingulate Gyrus, 12.7% Anterior Cingulate, 8% Medial Frontal Gyrus, 7.8% Middle Frontal Gyrus, 2.7% Superior Frontal Gyrus  60% Medial Frontal Gyrus, 24.8% Superior Frontal Gyrus, 15.1% Anterior Cingulate |
| ***Supplementary Table 4:*** *Detailed data and statistical figures for the 7 sub-analyses conducted for a family-wise corrected cluster level significance of p<0.05, and voxel wise threshold permutation of p<0.05, including: Contributing foci number, Number of Highlighted clusters, Centred MNI coordinates for the Clusters, Volume in mm^3 for the respective clusters, and the % contribution of the gyri corresponding to the clusters.*  *HC: Healthy Controls, CPP: Chronic Pain Patients, ICD-11: International Classification of Diseases-11, Chronic MSK Pain: Chronic Musculoskeletal Pain* | | | | | | | |

## **Supplementary Table 5:**

**Detailed data and statistical figures for the post-hoc analysis conducted for the primary analysis**

| **Sub analysis** | **Subcategories** | **Contrast** | **Contributing**  **Foci** | **Clusters** | **Centred MNI Coordinates** | **Volume** | **Gyrus** |
| --- | --- | --- | --- | --- | --- | --- | --- |
| **Chronic Pain** | Chronic Pain | HC > CPP | 488 | 4 | Cluster 1: 1.4, -53.9, 28  Cluster 2: 5.9, 58.5, 18.3  Cluster 3: 50.8, -58.8, 38.2  Cluster 4: -18.4, 55.7, 10.1 | 2416 mm^^3^  120 mm^^3^  864 mm^^3^  704 mm^^3^ | 55% Posterior Cingulate, 28.6% Cingulate Gyrus, 16.4% Precuneus  61.4% Medial Frontal Gyrus, 38.6% Superior Frontal Gyrus  1.4% Angular Gyrus, 14.3% Middle Temporal Gyrus, 14.3% Inferior Parietal Lobule  100% Superior Frontal Gyrus |
| ***Supplementary Table 5:*** *Detailed data and statistical figures for the post-hoc analysis conducted for the primary analysis (Chronic pain HC>CPP) at a threshold of voxel height, P<0.001 and familywise error–corrected cluster significance, P<0.05 including: Contributing foci number, Number of Highlighted clusters, Centred MNI coordinates for the Clusters, Volume in mm^3 for the respective clusters, and the % contribution of the gyri corresponding to the clusters.*  *HC: Healthy Controls, CPP: Chronic Pain Patients* | | | | | | | |

## **Supplementary Document 1**

The following supplementary document is the statistical data obtained for the 7 sub-analyses used in the study. The data was obtained using BrainMap Ginger ALE 2.3.6 alongside a pre-generated Python Code (Supplementary Document 2) to provide clusters corresponding to ALEs from the extracted MNI coordinates. This data is provided to exemplify Figure 3, description C.

1. Primary Meta – Analysis: Chronic Pain

Mask:

Reference Space = MNI

Dimensions = 77x96x79

Number of within-brain voxels = 229781

Mask Size = More Conservative (Smaller)

Foci:

Coordinate System = MNI

File of foci coordinates = CHRONIC PAIN WHOLE HC > P .txt

Number of foci = 488

Number of experiments = 61

Total number of subjects = 1749

ALE - Random Effects, Turkeltaub Non-Additive (HBM, 2012):

File of ALE voxels = CHRONIC PAIN WHOLE HC > P _ALE.nii

FWHM minimum value = 8.59081031745781

FWHM median value = 9.081322119956662

FWHM maximum value = 10.002568195473065

Minimum ALE score = 2.6418507E-18

Maximum ALE score = 0.027299635

P Values: Eickhoff (HBM, 2009)

File of P values = CHRONIC PAIN WHOLE HC > P _P.nii

Minimum P value = 6.837943E-7

Thresholding:

Threshold Method = Cluster-level Inference

Thresholding Value = 0.05

Thresholding Permutations = 1000

Cluster-Forming Method = Uncorrected P value

Cluster-Forming Value = 0.05

Volume > Threshold = 41768 mm^3

Chosen min. cluster size = 7680 mm^3

Thresholded ALE image = CHRONIC PAIN WHOLE HC > P _C05_1k_ALE.nii

Cluster Analysis:

#1: 16904 mm^3 from (-44,30,-12) to (22,70,40) centered at (-7.1,52.9,13.5) with 14 peaks

with a max value of 0.0273 ALE, 6.837943E-7 P, 4.83 Z at (6,56,16)

Labels: (Gray Matter only)

Hemisphere: 58.6% Left Cerebrum, 41.4% Right Cerebrum

Lobe: 75.1% Frontal Lobe, 24.9% Limbic Lobe

Gyrus: 52.7% Medial Frontal Gyrus, 24.9% Anterior Cingulate, 19.8% Superior Frontal Gyrus, 2.5% Middle Frontal Gyrus

Cell Type: 52.1% Brodmann area 9, 21.1% Brodmann area 10, 19.5% Brodmann area 32, 2.4% Brodmann area 24, 1.7% Brodmann area 8

#2: 14560 mm^3 from (-42,-2,-26) to (44,28,8) centered at (0.6,13.1,-4.8) with 11 peaks

with a max value of 0.0259 ALE, 1.6547576E-6 P, 4.65 Z at (12,12,-10)

Labels: (Gray Matter only)

Hemisphere: 51.7% Left Cerebrum, 48.3% Right Cerebrum

Lobe: 91.4% Sub-lobar, 5.1% Limbic Lobe, 3.4% Frontal Lobe

Gyrus: 51.6% Lentiform Nucleus, 22.9% Caudate, 6.4% Insula, 6.2% Claustrum, 5.1% Anterior Cingulate, 2% Inferior Frontal Gyrus, 1% Medial Frontal Gyrus

Cell Type: 42.4% Putamen, 20.1% Caudate Head, 8.2% Lateral Globus Pallidus, 6.9% Brodmann area 13, 4.5% Brodmann area 25, 2.8% Caudate Body, 1.4% Brodmann area 32

#3: 10304 mm^3 from (-16,-68,10) to (30,-42,42) centered at (2.6,-53.1,26.7) with 5 peaks

with a max value of 0.0224 ALE, 1.3813448E-5 P, 4.19 Z at (2,-56,26)

Labels: (Gray Matter only)

Hemisphere: 63.8% Left Cerebrum, 36.2% Right Cerebrum

Lobe: 64% Limbic Lobe, 28.3% Parietal Lobe, 7.7% Occipital Lobe

Gyrus: 43.9% Posterior Cingulate, 36% Precuneus, 20.2% Cingulate Gyrus

Cell Type: 59.4% Brodmann area 31, 19.7% Brodmann area 23, 11.4% Brodmann area 30, 5.9% Brodmann area 7, 3.5% Brodmann area 29

Experiment Table:

[ 0 3 0 0 0 0 3 0 0 0 1 0 2 0 0 2 1 0 3 0 0 0 0 1 0 0 0 1 0 2 1 1 0 1 0 0 0 0 1 0 0 8 0 0 0 0 0 0 1 0 2 2 1 0 5 4 2 0 1 0 0 ]

[ 0 0 0 0 0 0 0 0 0 0 0 0 2 0 0 0 0 0 0 0 0 0 0 0 0 0 0 2 0 0 1 0 0 0 0 0 0 0 5 0 3 0 0 4 2 1 1 3 0 2 1 0 0 0 11 4 2 0 1 0 0 ]

[ 0 0 0 0 0 0 0 0 0 0 0 1 0 0 6 1 0 0 2 0 0 1 0 4 0 0 0 0 0 0 0 0 0 1 0 0 0 0 0 0 2 5 0 0 0 0 0 2 1 0 1 0 0 0 2 2 1 0 0 0 0 ]

Contributors to cluster #1

3 foci from Wu, 2016: Controls>Patients

3 foci from Wang, 2017: Controls>Patients

1 foci from Ao, 2021: Controls>Patients

2 foci from Ke, 2015: Controls>Patients

2 foci from Dai, 2020: Controls>Patients  12 15 33

1 foci from Gu, 2019: Controls>Patients

3 foci from Cao, 2017: Controls>Patients

1 foci from Ihara, 2019: Controls>Patients

1 foci from Lin, 2017: Controls>Patients

2 foci from Park, 2020: Controls>Patients

1 foci from Pujol, 2014: Controls>Patients

1 foci from Kaplan, 2019: Controls>Patients

1 foci from Zou, 2021: Controls>Patients

1 foci from Gao, 2016: Controls>Patients

8 foci from Yong, 2016: Controls>Patients

1 foci from Qui, 2012: Controls>Patients

2 foci from Tetreault, 2018: Controls>Patients

2 foci from Lan, 2020: Controls>Patients

1 foci from Zhao, 2016: Controls>Patients

5 foci from Zhang, 2019: Controls>Patients

4 foci from Zhou, 2019: Controls>Patients

2 foci from Liu, 2020: Controls>Patients

1 foci from Zhou, 2018: Controls>Patients

Contributors to cluster #2

2 foci from Ke, 2015: Controls>Patients

2 foci from Lin, 2017: Controls>Patients

1 foci from Pujol, 2014: Controls>Patients

5 foci from Gao, 2016: Controls>Patients

3 foci from Zhao, 2017: Controls>Patients

4 foci from Zhao, 2014: Controls>Patients

2 foci from Chen, 2019: Controls>Patients

1 foci from Chou, 2017: Controls>Patients

1 foci from Chen, 2017: Controls>Patients

3 foci from Wang, 2014: Controls>Patients

2 foci from Barosso, 2021: Controls>Patients

1 foci from Tetreault, 2018: Controls>Patients

11 foci from Zhang, 2019: Controls>Patients

4 foci from Zhou, 2019: Controls>Patients

2 foci from Liu, 2020: Controls>Patients

1 foci from Zhou, 2018: Controls>Patients

Contributors to cluster #3

1 foci from Chen, 2021: Controls>Patients

6 foci from Huang, 2020: Controls>Patients

1 foci from Dai, 2020: Controls>Patients  12 15 33

2 foci from Cao, 2017: Controls>Patients

1 foci from Yue, 2020: Controls>Patients

4 foci from Ihara, 2019: Controls>Patients

1 foci from Zou, 2021: Controls>Patients

2 foci from Zhao, 2017: Controls>Patients

5 foci from Yong, 2016: Controls>Patients

2 foci from Wang, 2014: Controls>Patients

1 foci from Qui, 2012: Controls>Patients

1 foci from Tetreault, 2018: Controls>Patients

2 foci from Zhang, 2019: Controls>Patients

2 foci from Zhou, 2019: Controls>Patients

1. foci from Liu, 2020: Controls>Patients
2. Secondary Sub-analysis 1: ICD-11 Diagnosis Type
   1. Chronic Musculoskeletal Pain

Mask:

Reference Space = MNI

Dimensions = 77x96x79

Number of within-brain voxels = 229781

Mask Size = More Conservative (Smaller)

Foci:

Coordinate System = MNI

File of foci coordinates = Chronic MSK pain HC > P.txt

Number of foci = 142

Number of experiments = 16

Total number of subjects = 553

ALE - Random Effects, Turkeltaub Non-Additive (HBM, 2012):

File of ALE voxels = Chronic MSK pain HC > P_ALE.nii

FWHM minimum value = 8.59081031745781

FWHM median value = 9.081322119956662

FWHM maximum value = 9.868515026564658

Minimum ALE score = 9.2E-44

Maximum ALE score = 0.024616752

P Values: Eickhoff (HBM, 2009)

File of P values = Chronic MSK pain HC > P_P.nii

Minimum P value = 1.3348004E-8

Thresholding:

Threshold Method = Cluster-level Inference

Thresholding Value = 0.05

Thresholding Permutations = 1000

Cluster-Forming Method = Uncorrected P value

Cluster-Forming Value = 0.05

Volume > Threshold = 26968 mm^3

Chosen min. cluster size = 7304 mm^3

Thresholded ALE image = Chronic MSK pain HC > P_C05_1k_ALE.nii

Cluster Analysis:

#1: 10784 mm^3 from (-14,-70,20) to (14,-32,64) centered at (0,-51.4,34.2) with 10 peaks

with a max value of 0.0157 ALE, 1.614107E-5 P, 4.16 Z at (-2,-56,26)

Labels: (Gray Matter only)

Hemisphere: 69.4% Left Cerebrum, 30.6% Right Cerebrum

Lobe: 46.8% Limbic Lobe, 45.8% Parietal Lobe, 7.4% Occipital Lobe

Gyrus: 53% Precuneus, 25.2% Cingulate Gyrus, 21.6% Posterior Cingulate

Cell Type: 54.9% Brodmann area 31, 28.7% Brodmann area 7, 15.2% Brodmann area 23

#2: 8704 mm^3 from (2,-4,-22) to (42,26,6) centered at (19.3,11.6,-5.7) with 9 peaks

with a max value of 0.0152 ALE, 2.4744886E-5 P, 4.06 Z at (10,12,-10)

Labels: (Gray Matter only)

Hemisphere: 100% Right Cerebrum

Lobe: 94.4% Sub-lobar, 4.4% Limbic Lobe, 1.2% Frontal Lobe

Gyrus: 42.7% Lentiform Nucleus, 36.8% Caudate, 8.7% Claustrum, 4.4% Anterior Cingulate, 1.2% Subcallosal Gyrus

Cell Type: 32% Caudate Head, 30.8% Putamen, 8.2% Lateral Globus Pallidus, 4.8% Caudate Body, 4.2% Brodmann area 25, 3.8% Medial Globus Pallidus

#3: 7480 mm^3 from (-34,40,-14) to (2,68,24) centered at (-16.3,53.8,8.9) with 5 peaks

with a max value of 0.0246 ALE, 1.3348004E-8 P, 5.56 Z at (-18,56,10)

Labels: (Gray Matter only)

Hemisphere: 98.9% Left Cerebrum, 1.1% Right Cerebrum

Lobe: 50.3% Frontal Lobe, 49.7% Limbic Lobe

Gyrus: 49.7% Anterior Cingulate, 27.1% Medial Frontal Gyrus, 18.1% Superior Frontal Gyrus, 5.1% Middle Frontal Gyrus

Cell Type: 36.7% Brodmann area 32, 31.1% Brodmann area 10, 21.5% Brodmann area 9, 6.8% Brodmann area 24

Experiment Table:

[ 2 0 6 0 0 0 0 0 4 2 1 0 0 0 0 1 ]

[ 0 0 0 0 0 0 2 0 6 3 1 0 1 0 0 1 ]

[ 0 0 1 0 0 0 0 1 2 4 2 0 1 0 0 0 ]

Contributors to cluster #1

2 foci from Reference = MNI Yue, 2020: Controls>Patients

6 foci from Ihara, 2020: Controls>Patients

4 foci from Zhang, 2019: Controls>Patients

2 foci from Zhou, 2019: Controls>Patients

1 foci from Liu, 2020: Controls>Patients

1 foci from Tetreault, 2018: Controls>Patients

Contributors to cluster #2

2 foci from Barosso, 2021: Controls>Patients

6 foci from Zhang, 2019: Controls>Patients

3 foci from Zhou, 2019: Controls>Patients

1 foci from Liu, 2020: Controls>Patients

1 foci from Zhou, 2018: Controls>Patients

1 foci from Tetreault, 2018: Controls>Patients

Contributors to cluster #3

1 foci from Ihara, 2020: Controls>Patients

1 foci from Lan, 2020: Controls>Patients

2 foci from Zhang, 2019: Controls>Patients

4 foci from Zhou, 2019: Controls>Patients

2 foci from Liu, 2020: Controls>Patients

1. foci from Zhou, 2018: Controls>Patients
   1. Chronic Headache

Mask:

Reference Space = MNI

Dimensions = 77x96x79

Number of within-brain voxels = 229781

Mask Size = More Conservative (Smaller)

Foci:

Coordinate System = MNI

File of foci coordinates = Migraine - HC > P .txt

Number of foci = 158

Number of experiments = 17

Total number of subjects = 461

ALE - Random Effects, Turkeltaub Non-Additive (HBM, 2012):

File of ALE voxels = Migraine - HC > P _ALE.nii

FWHM minimum value = 8.781781412468805

FWHM median value = 9.056468275459084

FWHM maximum value = 10.002568195473065

Minimum ALE score = 4.646781E-25

Maximum ALE score = 0.01628238

P Values: Eickhoff (HBM, 2009)

File of P values = Migraine - HC > P _P.nii

Minimum P value = 1.0296578E-5

Thresholding:

Threshold Method = Cluster-level Inference

Thresholding Value = 0.05

Thresholding Permutations = 1000

Cluster-Forming Method = Uncorrected P value

Cluster-Forming Value = 0.05

Volume > Threshold = 6784 mm^3

Chosen min. cluster size = 5192 mm^3

Thresholded ALE image = Migraine - HC > P _C05_1k_ALE.nii

Cluster Analysis:

#1: 6784 mm^3 from (-18,-62,10) to (18,-40,42) centered at (2.8,-52.5,27.5) with 5 peaks

with a max value of 0.0109 ALE, 4.6422431E-4 P, 3.31 Z at (8,-52,34)

Labels: (Gray Matter only)

Hemisphere: 61.6% Right Cerebrum, 38.4% Left Cerebrum

Lobe: 66% Limbic Lobe, 28% Parietal Lobe, 6% Occipital Lobe

Gyrus: 41.4% Posterior Cingulate, 35.1% Precuneus, 23.5% Cingulate Gyrus

Cell Type: 72% Brodmann area 31, 15.7% Brodmann area 23, 6% Brodmann area 30, 3.7% Brodmann area 29, 2.6% Brodmann area 7

Experiment Table:

[ 0 1 0 0 0 0 0 0 2 5 0 0 0 0 2 1 0 ]

Contributors to cluster #1

1 foci from Zou, 2021: Controls>Migraine

2 foci from Zhao, 2017: Controls>Migraine

5 foci from Yong, 2016: Controls>Migraine

2 foci from Wang, 2014: Controls>Migraine

1. foci from Qui, 2012: Controls>Migraine
   1. Chronic Orofacial Pain

Mask:

Reference Space = MNI

Dimensions = 77x96x79

Number of within-brain voxels = 229781

Mask Size = More Conservative (Smaller)

Foci:

Coordinate System = MNI

File of foci coordinates = Orofacial HC > P .txt

Number of foci = 44

Number of experiments = 7

Total number of subjects = 167

ALE - Random Effects, Turkeltaub Non-Additive (HBM, 2012):

File of ALE voxels = Orofacial HC > P _ALE.nii

FWHM minimum value = 8.908865767289859

FWHM median value = 9.137264222884284

FWHM maximum value = 9.282869490900032

Minimum ALE score = 1.4E-45

Maximum ALE score = 0.009428888

P Values: Eickhoff (HBM, 2009)

File of P values = Orofacial HC > P _P.nii

Minimum P value = 5.788535E-5

Thresholding:

Threshold Method = Cluster-level Inference

Thresholding Value = 0.05

Thresholding Permutations = 1000

Cluster-Forming Method = Uncorrected P value

Cluster-Forming Value = 0.05

Volume > Threshold = 0 mm^3

Chosen min. cluster size = 8288 mm^3

Thresholded ALE image = Orofacial HC > P _C05_1k_ALE.nii

Cluster Analysis:

No clusters found

- 1. Chronic Visceral Pain

Mask:

Reference Space = MNI

Dimensions = 77x96x79

Number of within-brain voxels = 229781

Mask Size = More Conservative (Smaller)

Foci:

Coordinate System = MNI

File of foci coordinates = Chronic Visceral Pain HC > P .txt

Number of foci = 39

Number of experiments = 8

Total number of subjects = 219

ALE - Random Effects, Turkeltaub Non-Additive (HBM, 2012):

File of ALE voxels = Chronic Visceral Pain HC > P _ALE.nii

FWHM minimum value = 8.759548511260867

FWHM median value = 9.056468275459084

FWHM maximum value = 9.574940482272122

Minimum ALE score = 1.4E-45

Maximum ALE score = 0.011869384

P Values: Eickhoff (HBM, 2009)

File of P values = Chronic Visceral Pain HC > P _P.nii

Minimum P value = 1.6541719E-5

Thresholding:

Threshold Method = Cluster-level Inference

Thresholding Value = 0.05

Thresholding Permutations = 1000

Cluster-Forming Method = Uncorrected P value

Cluster-Forming Value = 0.05

Volume > Threshold = 23024 mm^3

Chosen min. cluster size = 7376 mm^3

Thresholded ALE image = Chronic Visceral Pain HC > P _C05_1k_ALE.nii

Cluster Analysis:

#1: 14200 mm^3 from (-38,4,10) to (16,52,42) centered at (-7.5,25.8,26.5) with 5 peaks

with a max value of 0.0119 ALE, 1.6541719E-5 P, 4.15 Z at (6,14,33)

Labels: (Gray Matter only)

Hemisphere: 69.3% Left Cerebrum, 30.7% Right Cerebrum

Lobe: 74.6% Limbic Lobe, 25.4% Frontal Lobe

Gyrus: 68.8% Cingulate Gyrus, 12.7% Anterior Cingulate, 8% Medial Frontal Gyrus, 7.8% Middle Frontal Gyrus, 2.7% Superior Frontal Gyrus

Cell Type: 42.5% Brodmann area 24, 35.4% Brodmann area 32, 12.9% Brodmann area 9, 6.4% Brodmann area 10, 2.9% Brodmann area 33

#2: 8824 mm^3 from (-14,42,-4) to (14,72,30) centered at (1.6,58.4,13.3) with 4 peaks

with a max value of 0.0106 ALE, 3.6883008E-5 P, 3.96 Z at (6,62,22)

Labels: (Gray Matter only)

Hemisphere: 53.1% Left Cerebrum, 46.9% Right Cerebrum

Lobe: 84.9% Frontal Lobe, 15.1% Limbic Lobe

Gyrus: 60% Medial Frontal Gyrus, 24.8% Superior Frontal Gyrus, 15.1% Anterior Cingulate

Cell Type: 52.5% Brodmann area 9, 33.3% Brodmann area 10, 14.3% Brodmann area 32

Experiment Table:

[ 0 0 0 1 0 3 0 2 ]

[ 0 3 0 0 0 0 0 1 ]

Contributors to cluster #1

1 foci from Ao, 2021: Controls>Patients

3 foci from Ke, 2015: Controls>Patients

2 foci from Lin, 2017: Controls>Patients

Contributors to cluster #2

3 foci from Wu, 2016: Controls>Patients

1. foci from Lin, 2017: Controls>Patients
2. Secondary Sub-analysis 2: Chronic Pain Mechanisms
   1. Neuropathic

Mask:

Reference Space = MNI

Dimensions = 77x96x79

Number of within-brain voxels = 229781

Mask Size = More Conservative (Smaller)

Foci:

Coordinate System = MNI

File of foci coordinates = Neuropathic HC > P .txt

Number of foci = 182

Number of experiments = 18

Total number of subjects = 448

ALE - Random Effects, Turkeltaub Non-Additive (HBM, 2012):

File of ALE voxels = Neuropathic HC > P _ALE.nii

FWHM minimum value = 8.722232250122541

FWHM median value = 9.137264222884284

FWHM maximum value = 9.755397007122182

Minimum ALE score = 5.482507E-23

Maximum ALE score = 0.024038367

P Values: Eickhoff (HBM, 2009)

File of P values = Neuropathic HC > P _P.nii

Minimum P value = 3.468962E-8

Thresholding:

Threshold Method = Cluster-level Inference

Thresholding Value = 0.05

Thresholding Permutations = 1000

Cluster-Forming Method = Uncorrected P value

Cluster-Forming Value = 0.05

Volume > Threshold = 17376 mm^3

Chosen min. cluster size = 6096 mm^3

Thresholded ALE image = Neuropathic HC > P _C05_1k_ALE.nii

Cluster Analysis:

#1: 8912 mm^3 from (-16,-66,20) to (14,-44,56) centered at (-.4,-53,34.4) with 10 peaks

with a max value of 0.0123 ALE, 2.6402736E-4 P, 3.47 Z at (-4,-48,30)

Labels: (Gray Matter only)

Hemisphere: 66.4% Left Cerebrum, 33.6% Right Cerebrum

Lobe: 55.3% Parietal Lobe, 39.4% Limbic Lobe, 5.4% Occipital Lobe

Gyrus: 60.6% Precuneus, 24.7% Posterior Cingulate, 14.6% Cingulate Gyrus

Cell Type: 47.6% Brodmann area 31, 34.4% Brodmann area 7, 12.8% Brodmann area 23, 5.2% Brodmann area 30

#2: 8464 mm^3 from (-34,40,2) to (10,70,34) centered at (-12.9,52.8,15.5) with 8 peaks

with a max value of 0.024 ALE, 3.468962E-8 P, 5.39 Z at (-18,56,10)

Labels: (Gray Matter only)

Hemisphere: 84.5% Left Cerebrum, 15.5% Right Cerebrum

Lobe: 89% Frontal Lobe, 11% Limbic Lobe

Gyrus: 55.7% Medial Frontal Gyrus, 24.6% Superior Frontal Gyrus, 10.6% Anterior Cingulate, 8.7% Middle Frontal Gyrus

Cell Type: 81.1% Brodmann area 9, 10.2% Brodmann area 32, 8.3% Brodmann area 10

Experiment Table:

[ 0 0 0 0 0 6 1 0 0 0 0 2 0 0 0 4 2 0 ]

[ 0 0 0 0 3 0 0 1 0 2 0 1 0 0 1 2 4 1 ]

Contributors to cluster #1

6 foci from Huang, 2020: Controls>Patients

1 foci from Dai, 2020: Controls>Patients

2 foci from Yoshino, 2015: Controls>Patients

4 foci from Zhang, 2019: Controls>Patients

2 foci from Zhou, 2019: Controls>Patients

Contributors to cluster #2

3 foci from Wang, 2017: Controls>Patients

1 foci from Gu, 2019: Controls>Patients

2 foci from Cao, 2017: Controls>Patients

1 foci from Yoshino, 2015: Controls>Patients

1 foci from Park, 2020: Controls>Patients

2 foci from Zhang, 2019: Controls>Patients

4 foci from Zhou, 2019: Controls>Patients

1. foci from Zhou, 2018: Controls>Patients
   1. Nociceptive

Mask:

Reference Space = MNI

Dimensions = 77x96x79

Number of within-brain voxels = 229781

Mask Size = More Conservative (Smaller)

Foci:

Coordinate System = MNI

File of foci coordinates = Nociceptive HC > P .txt

Number of foci = 72

Number of experiments = 12

Total number of subjects = 456

ALE - Random Effects, Turkeltaub Non-Additive (HBM, 2012):

File of ALE voxels = Nociceptive HC > P _ALE.nii

FWHM minimum value = 8.59081031745781

FWHM median value = 9.081322119956662

FWHM maximum value = 9.755397007122182

Minimum ALE score = 1.4E-45

Maximum ALE score = 0.016502023

P Values: Eickhoff (HBM, 2009)

File of P values = Nociceptive HC > P _P.nii

Minimum P value = 1.6531694E-6

Thresholding:

Threshold Method = Cluster-level Inference

Thresholding Value = 0.05

Thresholding Permutations = 1000

Cluster-Forming Method = Uncorrected P value

Cluster-Forming Value = 0.05

Volume > Threshold = 13968 mm^3

Chosen min. cluster size = 6392 mm^3

Thresholded ALE image = Nociceptive HC > P _C05_1k_ALE.nii

Cluster Analysis:

#1: 13968 mm^3 from (-14,-72,8) to (24,-18,46) centered at (3.7,-46.4,28.4) with 9 peaks

with a max value of 0.0102 ALE, 1.8141221E-4 P, 3.57 Z at (8,-48,30)

Labels: (Gray Matter only)

Hemisphere: 52.9% Left Cerebrum, 47.1% Right Cerebrum

Lobe: 47.5% Limbic Lobe, 26.4% Parietal Lobe, 16.8% Sub-lobar, 9.4% Occipital Lobe

Gyrus: 34.8% Precuneus, 27.4% Cingulate Gyrus, 20.1% Posterior Cingulate, 16% Thalamus

Cell Type: 54.6% Brodmann area 31, 14.4% Brodmann area 23, 10.4% Brodmann area 7, 9.9% Pulvinar, 2.8% Lateral Posterior Nucleus, 2.6% Brodmann area 30

Experiment Table:

[ 0 2 5 0 0 0 0 1 0 1 0 2 ]

Contributors to cluster #1

2 foci from Yue, 2020: Controls>Patients

5 foci from Ihara, 2019: Controls>Patients

1 foci from Tetreault, 2018: Controls>Patients

1 foci from Liu, 2020: Controls>Patients

1. foci from Rogachov, 2018: Controls>Patients
   1. Nociplastic

Mask:

Reference Space = MNI

Dimensions = 77x96x79

Number of within-brain voxels = 229781

Mask Size = More Conservative (Smaller)

Foci:

Coordinate System = MNI

File of foci coordinates = Nociplastic HC > P .txt

Number of foci = 235

Number of experiments = 30

Total number of subjects = 820

ALE - Random Effects, Turkeltaub Non-Additive (HBM, 2012):

File of ALE voxels = Nociplastic HC > P _ALE.nii

FWHM minimum value = 8.759548511260867

FWHM median value = 9.108170585812841

FWHM maximum value = 10.002568195473065

Minimum ALE score = 4.848634E-25

Maximum ALE score = 0.021790903

P Values: Eickhoff (HBM, 2009)

File of P values = Nociplastic HC > P _P.nii

Minimum P value = 9.911504E-7

Thresholding:

Threshold Method = Cluster-level Inference

Thresholding Value = 0.05

Thresholding Permutations = 1000

Cluster-Forming Method = Uncorrected P value

Cluster-Forming Value = 0.05

Volume > Threshold = 19696 mm^3

Chosen min. cluster size = 5256 mm^3

Thresholded ALE image = Nociplastic HC > P _C05_1k_ALE.nii

Cluster Analysis:

#1: 7280 mm^3 from (-46,-4,-22) to (-18,40,18) centered at (-31.5,15,-.2) with 5 peaks

with a max value of 0.0218 ALE, 9.911504E-7 P, 4.76 Z at (-34,16,2)

Labels: (Gray Matter only)

Hemisphere: 100% Left Cerebrum

Lobe: 91% Sub-lobar, 6.2% Frontal Lobe, 1.7% Limbic Lobe, 1.1% Temporal Lobe

Gyrus: 66.9% Lentiform Nucleus, 13.2% Insula, 10.9% Claustrum, 5.9% Inferior Frontal Gyrus, 1.4% Parahippocampal Gyrus

Cell Type: 58.3% Putamen, 16.2% Brodmann area 13, 8.7% Lateral Globus Pallidus, 2.5% Brodmann area 45, 1.1% Brodmann area 34

#2: 6936 mm^3 from (-10,44,0) to (18,72,38) centered at (3.8,56.7,16.4) with 5 peaks

with a max value of 0.0196 ALE, 4.4129947E-6 P, 4.44 Z at (6,56,16)

Labels: (Gray Matter only)

Hemisphere: 58.2% Right Cerebrum, 41.8% Left Cerebrum

Lobe: 93.5% Frontal Lobe, 6.5% Limbic Lobe

Gyrus: 66.7% Medial Frontal Gyrus, 26.8% Superior Frontal Gyrus, 6.5% Anterior Cingulate

Cell Type: 65.3% Brodmann area 9, 28.2% Brodmann area 10, 6.5% Brodmann area 32

#3: 5480 mm^3 from (-18,-60,12) to (18,-42,40) centered at (1.8,-52.9,25.7) with 4 peaks

with a max value of 0.014 ALE, 2.0213178E-4 P, 3.54 Z at (-2,-54,18)

Labels: (Gray Matter only)

Hemisphere: 53.7% Right Cerebrum, 46.3% Left Cerebrum

Lobe: 73.6% Limbic Lobe, 22.9% Parietal Lobe, 3.5% Occipital Lobe

Gyrus: 53.3% Posterior Cingulate, 26.4% Precuneus, 20.3% Cingulate Gyrus

Cell Type: 62.1% Brodmann area 31, 18.9% Brodmann area 23, 11.9% Brodmann area 30, 4.8% Brodmann area 29, 2.2% Brodmann area 7

Experiment Table:

[ 0 0 0 0 0 0 0 1 0 1 1 0 0 0 1 0 0 0 5 0 1 0 0 0 1 1 1 0 0 0 ]

[ 0 3 2 0 0 0 0 0 0 1 0 1 0 0 0 0 0 0 0 0 0 5 0 0 0 0 0 1 1 0 ]

[ 0 0 0 0 0 0 1 0 0 0 0 0 0 1 0 0 0 0 0 0 2 5 0 0 0 0 2 1 0 0 ]

Contributors to cluster #1

1 foci from Ke, 2015: Controls>Patients

1 foci from Lin, 2017: Controls>Patients

1 foci from Pujol, 2014: Controls>Patients

1 foci from Zhengjie, 2020: Controls>Patients

5 foci from Gao, 2016: Controls>Patients

1 foci from Zhao, 2017: Controls>Patients

1 foci from Chen, 2019: Controls>Patients

1 foci from Chou, 2017: Controls>Patients

1 foci from Wang, 2014: Controls>Patients

Contributors to cluster #2

3 foci from Wu, 2016: Controls>Patients

2 foci from Lim, 2021: Controls>Patients

1 foci from Lin, 2017: Controls>Patients

1 foci from Kaplan, 2019: Controls>Patients

5 foci from Yong, 2016: Controls>Patients

1 foci from Qui, 2012: Controls>Patients

1 foci from Zhao, 2016: Controls>Patients

Contributors to cluster #3

1 foci from Chen, 2021: Controls>Patients

1 foci from Zou, 2021: Controls>Patients

2 foci from Zhao, 2017: Controls>Patients

5 foci from Yong, 2016: Controls>Patients

2 foci from Wang, 2014: Controls>Patients

1 foci from Qui, 2012: Controls>Patients

## **Supplementary Document 2**

The Following Python code was used to overlap previously published HCP parcellation on the constructed ALEs (generation depicted in Supplementary Document 1) to match ALEs to their corresponding parcellation. The generated 2-dimensional images were automatically generated by Python to visually represent the parcellations. This data is provided to exemplify Figure 3, description C.

Purpose of the code:

The purpose of this module is to enable comparison between Spherical clusters (defined by coordinates in MNI space and a radius) and the HCP Parcellation

This module compares the clusters to the parcellations spatially and determines

all the parcellations that overlap, and what percentage is overlapping

Code as below:

def sphere(shape, radius, position):

# assume shape and position are both a 3-tuple of int or float

# the units are pixels / voxels (px for short)

# radius is a int or float in px

semisizes = (radius,) * 3

# genereate the grid for the support points

# centered at the position indicated by position

grid = [slice(-x0, dim - x0) for x0, dim in zip(position, shape)]

position = np.ogrid[grid]

# calculate the distance of all points from `position` center

# scaled by the radius

arr = np.zeros(shape, dtype=float)

for x_i, semisize in zip(position, semisizes):

arr += (np.abs(x_i / semisize) ** 2)

# the inner part of the sphere will have distance below 1

return arr <= 1.0

def cluster_to_parc(cluster_coords, cluster_id=None, cluster_vol=None, output_path=None):

"""

Maps a set of MNI coordinates to the nearest HCP parcellations in the modified HCP-MMP-01

parcellation scheme

A sphere is placed at the MNI Coordinates with the radius specified.

The HCP MMP voxels that overlap with that sphere are calculated.

Stats on the % of each parcellation that falls into the sphere are returned

The Parcellation (cortical and subcortical) with the most overlap is designated as the

'primary parcellation'

Inputs:

Cluster_cords = Pandas DataFrame of x, y, z coords

Cluster_ID = (numpy array) optional ID corresponding to each set of cluster_cords

Cluster_vol = (numpy array) optional ID corresponding to the volume in mm^3

>> gets converted to radius in 2mm voxels of the sphere to generate

Output path = (path) If provided images saved to the path specified

"""

atlas = nib.load('/Users/isabellayoung/Desktop/glasser_subcortical_transformed.nii.gz') # TODO: edit path

mni = datasets.load_mni152_template()

aff = mni.affine

parc_df = pd.read_csv(**PATH_TO_ATLAS_CIRCUITS.CSV*) # TODO: edit path

full_cluster_df = pd.DataFrame()

primary_df = pd.DataFrame()

if output_path is not None:

Path(output_path).mkdir(parents=True, exist_ok=True)

for i in tqdm(range(len(cluster_coords))):

cluster = cluster_coords.iloc[i]

if cluster_vol is not None:

# Assumes radius column is given in mm^3

vol_mm3 = cluster[cluster_vol]

# Convert to radius in 2mm voxels of the sphere to generate (minus middle voxel)

r = (((3*vol_mm3) / (4*math.pi)) ** (1/3) - 1) /2

else:

# TODO: can adjust if no volume column

# E.g. if desired diameter is 15mm, then r = ((15/2)-1)/2 = 3.25

r = 3.25

# Extract x, y, z from MNI

x = cluster['x']

y = cluster['y']

z = cluster['z']

res = {

'MNI': str(x)+' '+str(y)+' '+str(z),

}

if cluster_id is not None:

res['Cluster ID'] = cluster_coords[cluster_id][i]

else:

res['Cluster ID'] = i

# Convert these to numpy coordinates

aff_inv = np.linalg.inv(aff)

x1, y1, z1 = [int(k) for k in nib.affines.apply_affine(aff_inv, ([x,y,z]))]

# sphere with radius generated

cluster_mask = sphere(mni.shape, r, [x1, y1, z1])

cluster_mask = np.where(cluster_mask,1,0)

cluster_img = nib.Nifti1Image(cluster_mask, aff)

#resample to mask

cluster_img_res = resample_to_img(cluster_img, atlas, 'nearest')

# Extract parcellations

cluster_mask = cluster_img_res.get_fdata()>0

parc_data = atlas.get_fdata()[cluster_mask]

parc_data = np.int_(np.round(parc_data))

parc_count = dict(Counter(parc_data[parc_data!=0]))

total = np.count_nonzero(parc_data+1)

parc_perc = {key:100*parc_count[key]/total for key in parc_count.keys()}

cluster_df = pd.DataFrame()

for key in parc_perc.keys():

# calculate precentage overlap

cluster_size_vox = np.count_nonzero(cluster_mask)

parc_size_vox = np.count_nonzero(np.int_(np.round(atlas.get_fdata())) == key)

overlap_vox = parc_count[key]

perc_overlap = overlap_vox / parc_size_vox

flat = deepcopy(res)

flat['parcellation key']=key

flat['parcellation name']=parc_df.loc[parc_df['ID']==key]['Old Name'].values[0]

flat['percentage overlap']=perc_overlap

full_cluster_df = full_cluster_df.append(flat, ignore_index=True)

cluster_df = cluster_df.append(flat, ignore_index=True)

if len(cluster_df) != 0:

idx_max_perc = cluster_df['percentage overlap'].idxmax()

max_parc_row = cluster_df.iloc[idx_max_perc]

max_key = max_parc_row['parcellation key']

max_val = max_parc_row['percentage overlap']

max_parc_name = max_parc_row['parcellation name']

primary = deepcopy(res)

primary['parcellation key'] = max_key

primary['percentage overlap'] = max_val

primary['parcellation name'] = max_parc_name

primary_df = primary_df.append(primary, ignore_index=True)

elif len(cluster_df) == 0:

primary = deepcopy(res)

primary['parcellation key'] = None

primary['percentage overlap'] = None

primary['parcellation name'] = None

primary_df = primary_df.append(primary, ignore_index=True)

if output_path is not None:

plot_output = output_path+'Cluster_'+str(res['Cluster ID'])

# Plot Cluster

plot = plotting.plot_roi(cluster_img_res, bg_img=mni, cut_coords=(x, y, z), draw_cross=False, cmap='bwr')

plot.title(res['Cluster ID'],x=0.01,y=0.10)

plot.savefig(plot_output+'.png')

plt.clf()

plt.close('all')

# Plot all clusters and Parc

for idx, row in cluster_df.iterrows():

parc_key = row['parcellation key']

parc_name = row['parcellation name']

# Generate Image with Cluster and Parc

parc_mask = np.where(np.int_(np.round(atlas.get_fdata()))==parc_key,2,cluster_img_res.get_fdata())

parc_mask_img= nib.Nifti1Image(parc_mask, cluster_img_res.affine)

# Plot cluster and Parc

plot = plotting.plot_roi(parc_mask_img, bg_img=mni, cut_coords=(x, y, z), draw_cross=False, cmap='bwr')

plot.title(str(res['Cluster ID'])+' - '+parc_name,x=0.01,y=0.10)

plot.savefig(plot_output+'_'+parc_name+'.png')

plt.clf()

plt.close('all')

if output_path is not None:

full_cluster_df.to_csv(output_path+'clusters_full.csv')

primary_df.to_csv(output_path+'primary_clusters.csv')

return full_cluster_df, primary_df

## **Supplementary Figure 1**

Supplementary Figures 1a-g are two-dimensional images of the brain that illustrates underactive parcellations for the contrast Healthy controls > Chronic Pain Patients (HC > CPP) for the respective ALEs. The images were generated automatically by the python code that generated the ALEs from the MNI coordinates. Alongside the parcellation names the MNI coordinates corresponding to the parcellations are provided on the figures (the Y coordinates from the MNI coordinates were concealed due to inevitable coding principles). To compensate for this, the data is summarized in the figures’ corresponding tables.

**Supplementary Figure 1a: Chronic Pain HC > CPP _ALE**

2-dimensional illustration of the 46 parcellations displaying underactivity in chronic pain patients in comparison to healthy controls across 3 major clusters.

*Display of Figures Divided into 3 Clusters*

**Supplementary Figure 1b: Chronic Headache HC > CPP _ALE**

2-dimensional illustration of the 13 parcellations displaying underactivity in chronic headache patients in comparison to healthy controls across 1 major cluster.

*Display of Figures as 1 Cluster*

**Supplementary Figure 1c: Chronic MSK Pain HC > CPP _ALE**

2-dimensional illustration of the 37 parcellations displaying underactivity in chronic musculoskeletal pain patients in comparison to healthy controls across 3 major clusters.

*Display of Figures Divided into 3 Clusters*

**Supplementary Figure 1d: Chronic Visceral Pain HC > CPP _ALE**

2-dimensional illustration of the 22 parcellations displaying underactivity in chronic visceral pain patients in comparison to healthy controls across 2 major clusters.

*Display of Figures Divided into 2 Clusters*

**Supplementary Figure 1e: Neuropathic HC > CPP _ALE**

2-dimensional illustration of the 24 parcellations displaying underactivity in chronic neuropathic pain patients in comparison to healthy controls across 2 major clusters.

*Display of Figures Divided into 2 Clusters*

**Supplementary Figure 1f: Nociplastic HC > CPP _ALE**

2-dimensional illustration of the 28 parcellations displaying underactivity in chronic nociplastic pain patients in comparison to healthy controls across 3 major clusters.

*Display of Figures Divided into 3 Clusters*

**Supplementary Figure 1g: Nociceptive HC > CPP _ALE**

2-dimensional illustration of the 16 parcellations displaying underactivity in chronic nociceptive pain patients in comparison to healthy controls across 1 major cluster.

*Display of Figures as 1 Cluster*

**Supplementary Figure 1a**

1.
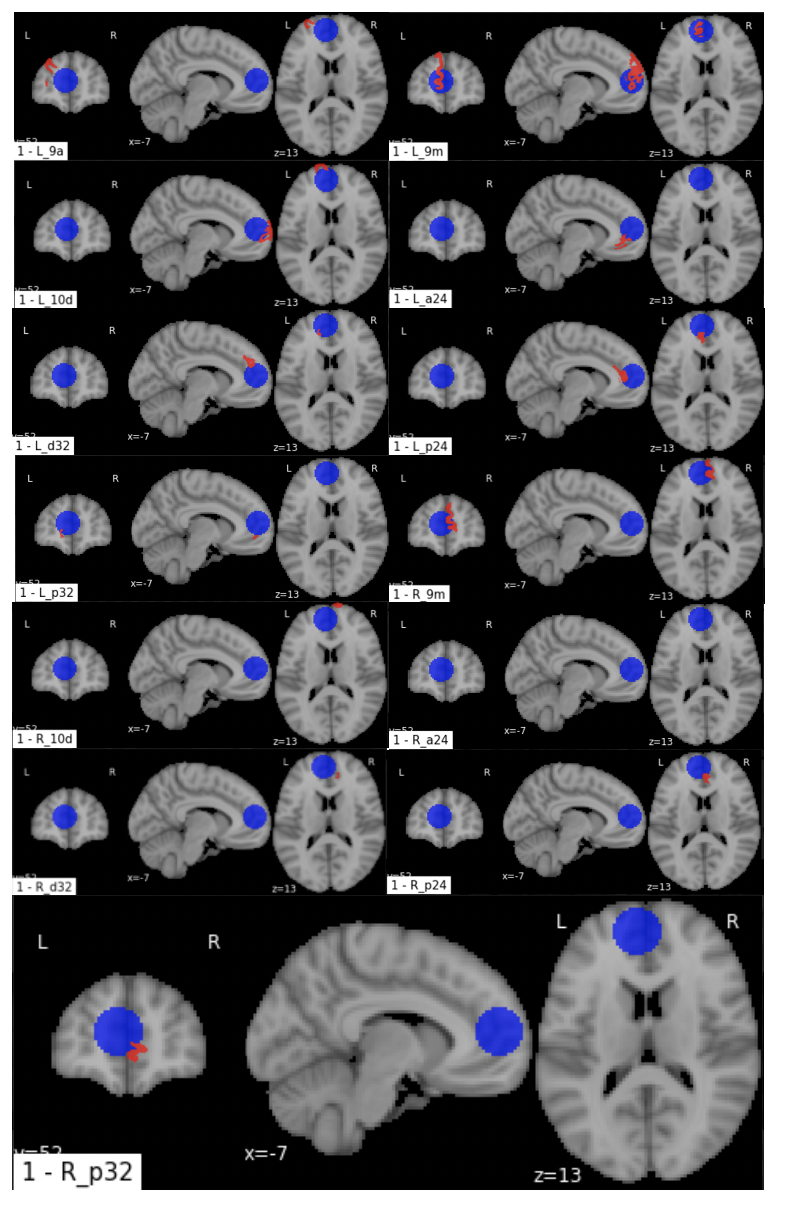
Cluster 1

**Supplementary Figure 1a**

1.
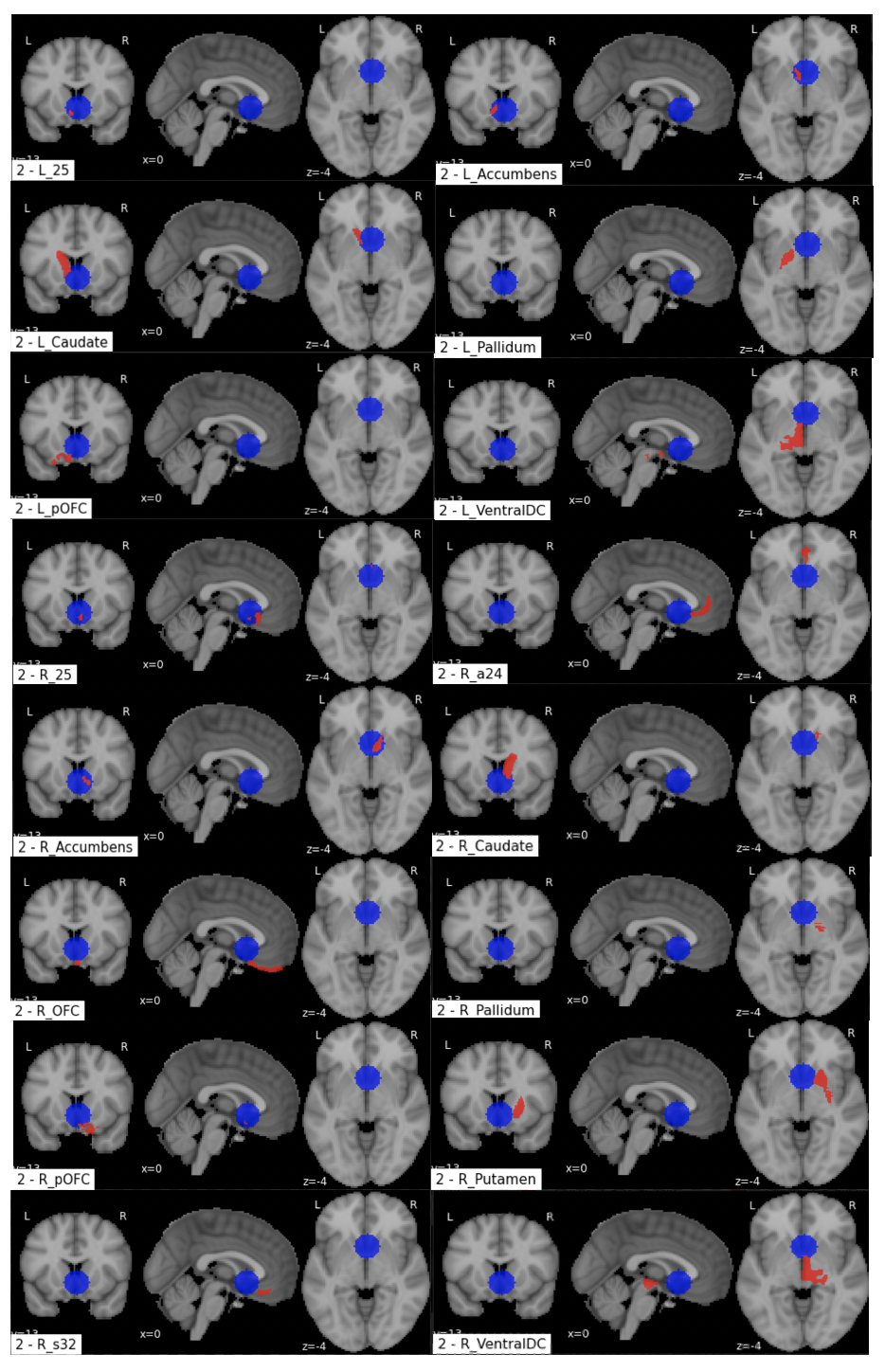
Cluster 2

**Supplementary Figure 1a**


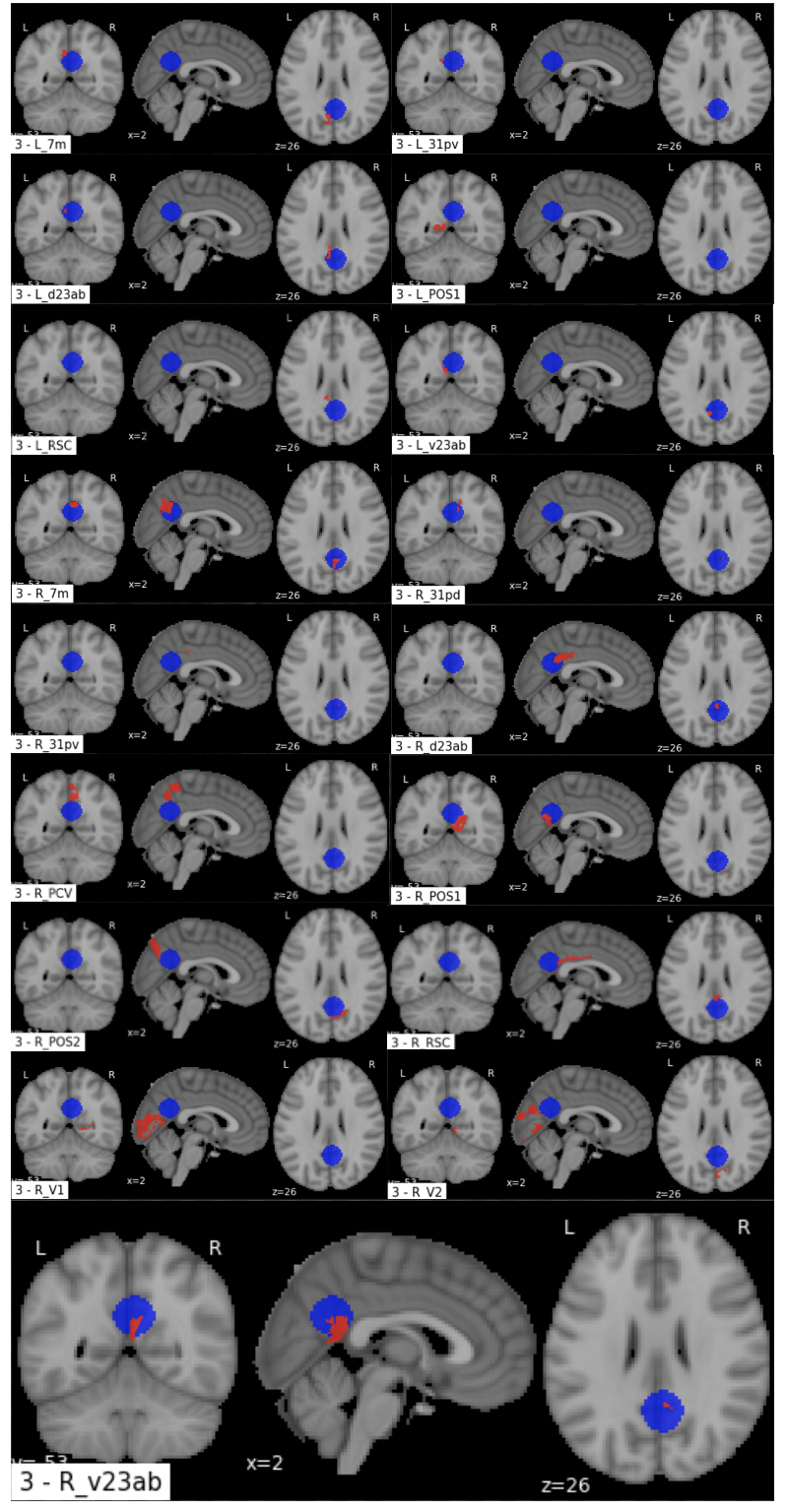


1. Cluster 3

**Supplementary Figure 1a**

1. Table corresponding to the 3 clusters for: **Chronic Pain HC > CPP _ALE**

|  | **Cluster ID** | **MNI Coordinates** | **Parcellation Name** |
| --- | --- | --- | --- |
|  | 1 | -7.1, 52.9, 13.5 | R_9m |
|  |  |  | R_a24 |
|  |  |  | R_p32 |
|  |  |  | R_p24 |
|  |  |  | R_d32 |
|  |  |  | R_10d |
|  |  |  | L_d32 |
|  |  |  | L_9m |
|  |  |  | L_p24 |
|  |  |  | L_a24 |
|  |  |  | L_10d |
|  |  |  | L_p32 |
|  |  |  | L_9a |
|  | 2 | 0.6, 13.1, -4.8 | R_Putamen |
|  |  |  | R_Caudate |
|  |  |  | R_Pallidum |
|  |  |  | R_Accumbens |
|  |  |  | R_pOFC |
|  |  |  | R_VentralDC |
|  |  |  | R_25 |
|  |  |  | R_s32 |
|  |  |  | R_OFC |
|  |  |  | R_a24 |
|  |  |  | L_VentralDC |
|  |  |  | L_pOFC |
|  |  |  | L_25 |
|  |  |  | L_Accumbens |
|  |  |  | L_Caudate |
|  |  |  | L_Pallidum |
|  | 3 | 2.6 -53.1 26.7 | R_POS1 |
|  |  |  | R_POS2 |
|  |  |  | R_31pv |
|  |  |  | R_31pd |
|  |  |  | R_7m |
|  |  |  | R_v23ab |
|  |  |  | R_d23ab |
|  |  |  | R_RSC |
|  |  |  | R_PCV |
|  |  |  | R_V2 |
|  |  |  | R_V1 |
|  |  |  | L_7m |
|  |  |  | L_d23ab |
|  |  |  | L_v23ab |
|  |  |  | L_POS1 |
|  |  |  | L_RSC |
|  |  |  | L_31pv |

**Supplementary Figure 1b**

*
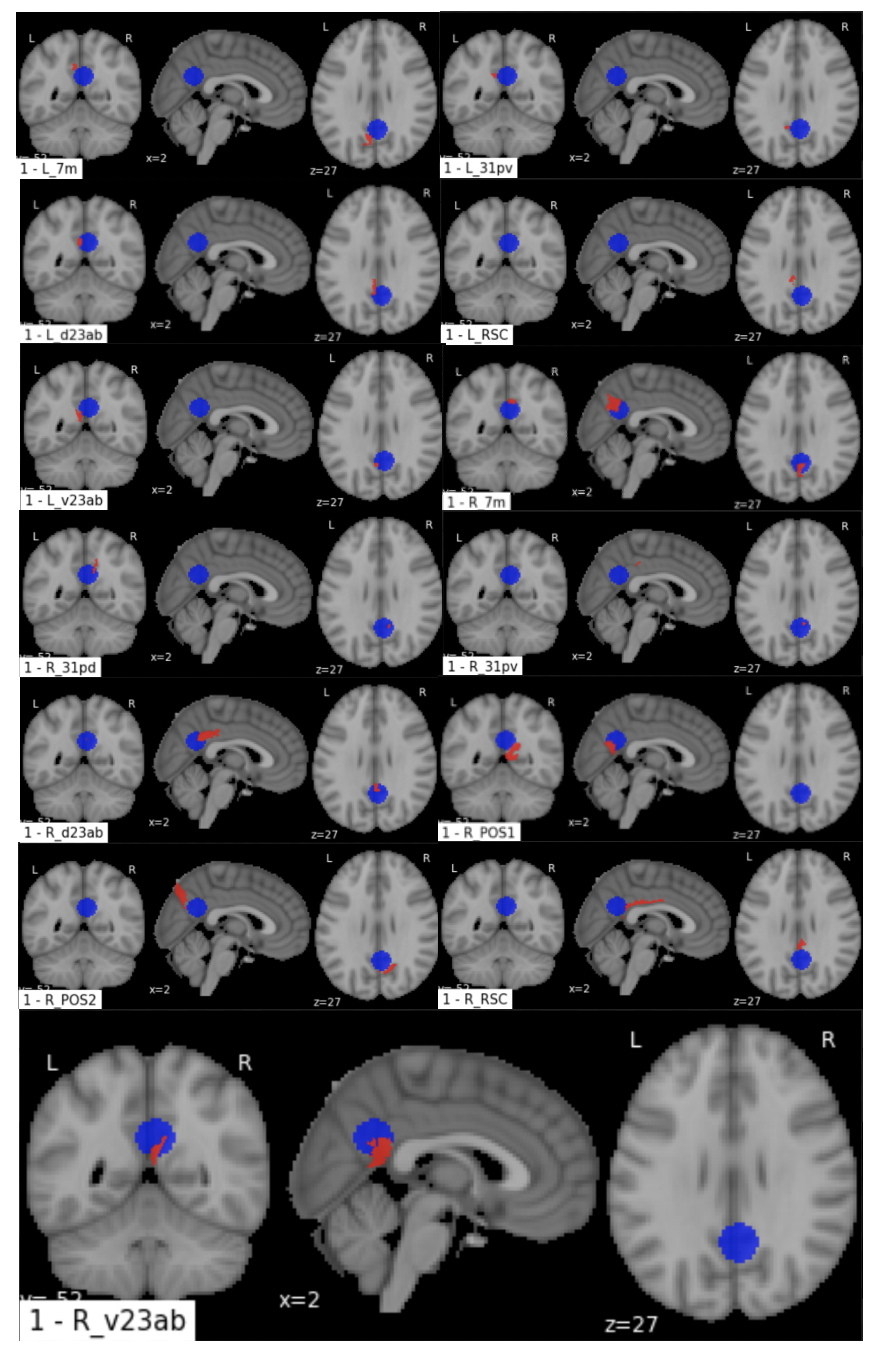
*

1. Cluster 1

**Supplementary Figure 1b**

1. Table corresponding to the 1 cluster for: **Chronic Headache HC > CPP _ALE**

|  | **Cluster ID** | **MNI Coordinates** | **Parcellation Name** |
| --- | --- | --- | --- |
|  | 1 | 2.8, -52.5, 27.5 | R_POS1 |
|  |  |  | R_31pd |
|  |  |  | R_31pv |
|  |  |  | R_POS2 |
|  |  |  | R_7m |
|  |  |  | R_v23ab |
|  |  |  | R_d23ab |
|  |  |  | R_RSC |
|  |  |  | L_7m |
|  |  |  | L_d23ab |
|  |  |  | L_v23ab |
|  |  |  | L_RSC |
|  |  |  | L_31pv |


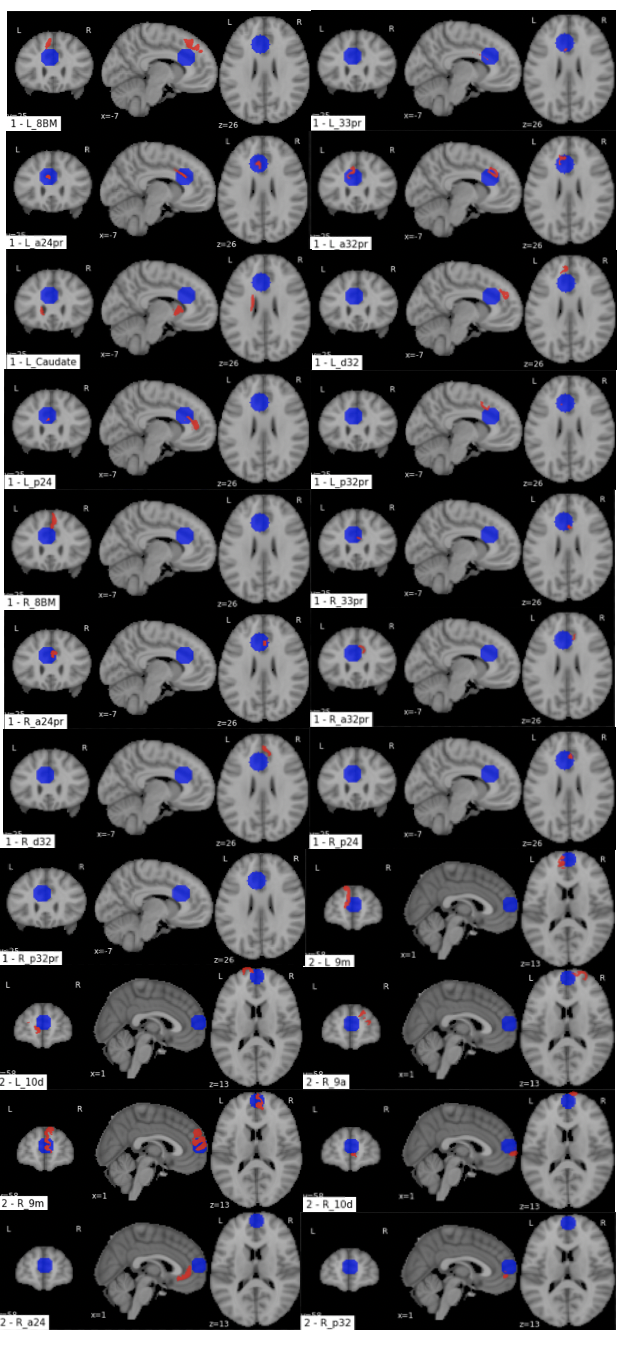


**Supplementary Figure 1c**

1. Cluster 1

**Supplementary Figure 1c**

1. Cluster 2


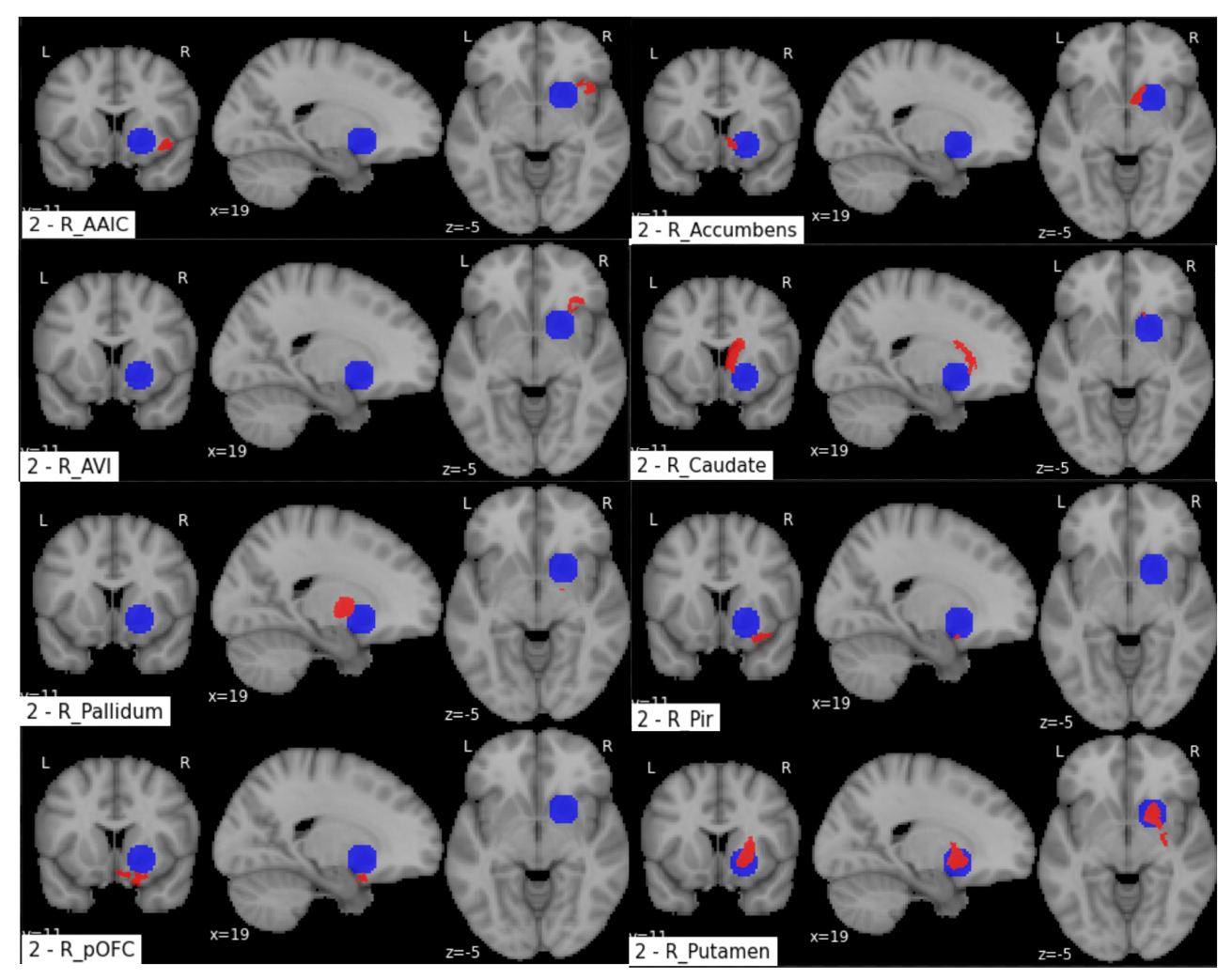


**Supplementary Figure 1c**

1. Cluster 3


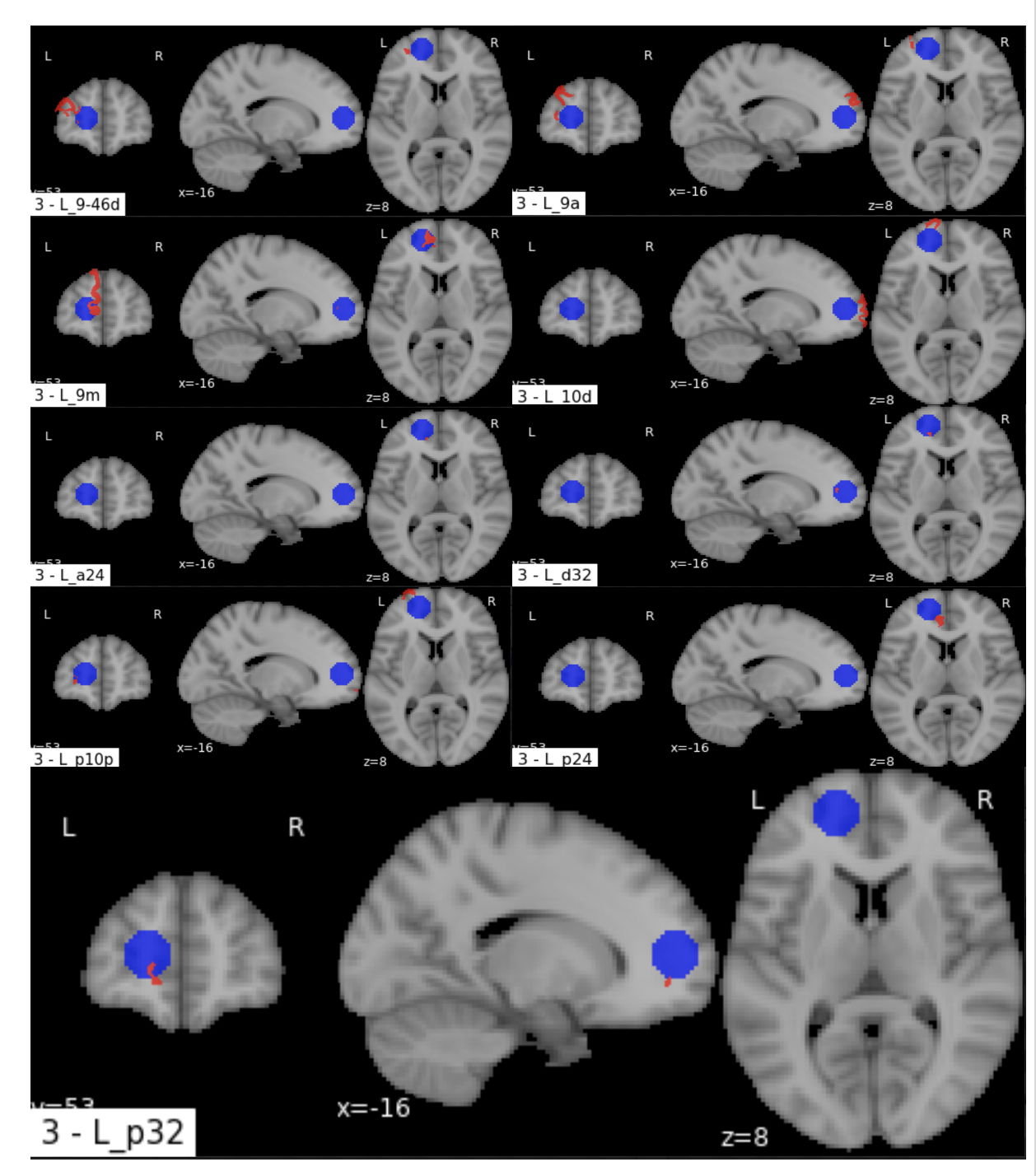


|  | **Cluster ID** | **MNI Coordinates** | **Parcellation Name** |
| --- | --- | --- | --- |
|  | 1 | 1. , -51.4, 34.2 | R_31pd |
|  |  |  | R_31pv |
|  |  |  | R_7m |
|  |  |  | R_31a |
|  |  |  | R_PCV |
|  |  |  | R_v23ab |
|  |  |  | R_d23ab |
|  |  |  | R_23c |
|  |  |  | R_RSC |
|  |  |  | R_POS2 |
|  |  |  | R_POS1 |
|  |  |  | L_PCV |
|  |  |  | L_7m |
|  |  |  | L_POS2 |
|  |  |  | L_d23ab |
|  |  |  | L_v23ab |
|  |  |  | L_RSC |
|  |  |  | L_31pv |
|  |  |  | L_31a |
|  |  |  | L_31pd |
|  | 2 | 19.3, 11.6, -5.7 | R_Putamen |
|  |  |  | R_AVI |
|  |  |  | R_AAIC |
|  |  |  | R_Pir |
|  |  |  | R_Pallidum |
|  |  |  | R_Caudate |
|  |  |  | R_pOFC |
|  |  |  | R_Accumbens |
|  | 3 | -16.3, 53.8, 8.9 | L_9m |
|  |  |  | L_p24 |
|  |  |  | L_10d |
|  |  |  | L_a24 |
|  |  |  | L_p32 |
|  |  |  | L_d32 |
|  |  |  | L_9a |
|  |  |  | L_9-46d |
|  |  |  | L_p10p |

**Supplementary Figure 1c**

1. Table corresponding to the 3 clusters for: **Chronic MSK Pain HC > CPP _ALE**

**
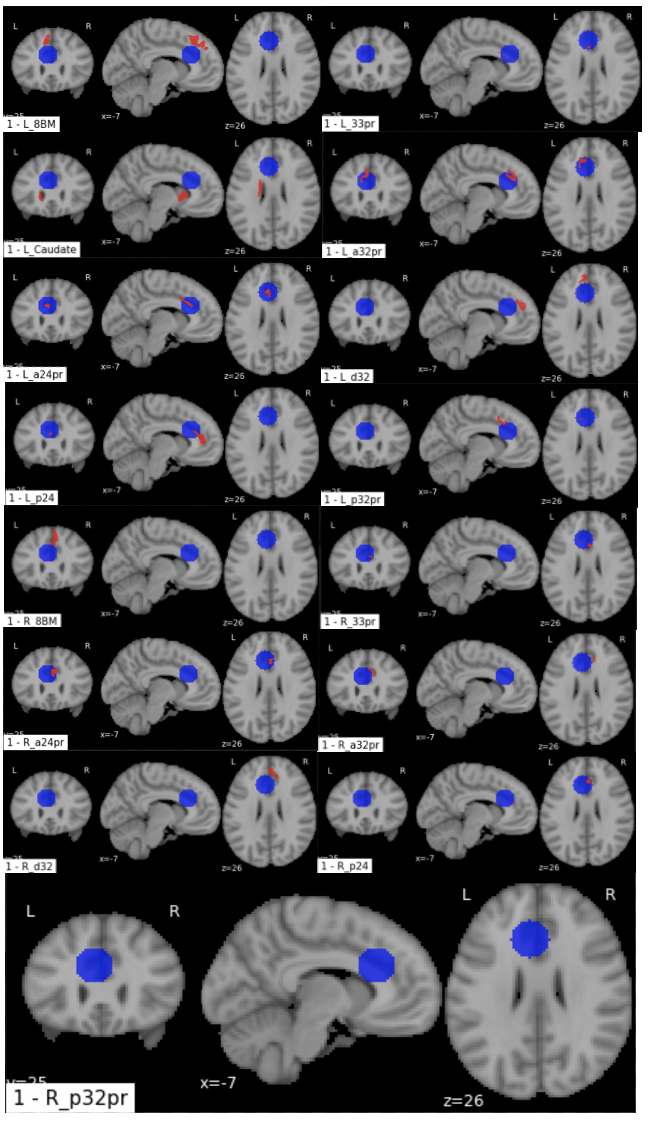
Supplementary Figure 1d**

1. Cluster 1

**Supplementary Figure 1d**

1. Cluster 2


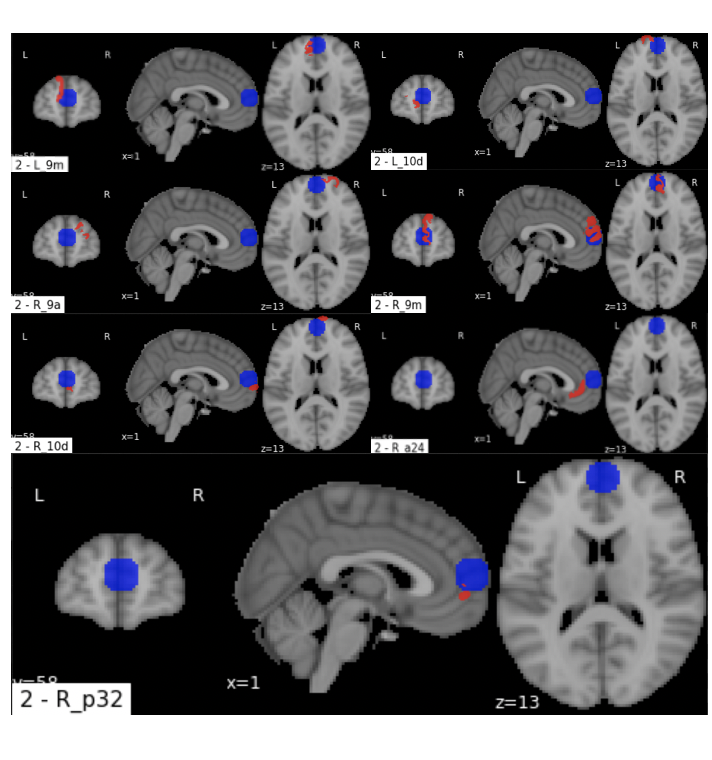


**Supplementary Figure 1d**

1. Table corresponding to the 2 clusters for: **Chronic Visceral Pain HC > CPP _ALE**

|  | **Cluster ID** | **MNI Coordinates** | **Parcellation Name** |
| --- | --- | --- | --- |
|  | 1 | -7.5, 25.8, 26.5 | R_a24pr |
|  |  |  | R_p32pr |
|  |  |  | R_d32 |
|  |  |  | R_a32pr |
|  |  |  | R_p24 |
|  |  |  | R_33pr |
|  |  |  | R_8BM |
|  |  |  | L_p24 |
|  |  |  | L_a32pr |
|  |  |  | L_a24pr |
|  |  |  | L_33pr |
|  |  |  | L_p32pr |
|  |  |  | L_d32 |
|  |  |  | L_8BM |
|  |  |  | L_Caudate |
|  | 2 | 1.6, 58.4, 13.3 | R_9a |
|  |  |  | R_9m |
|  |  |  | R_10d |
|  |  |  | R_p32 |
|  |  |  | R_a24 |
|  |  |  | L_9m |
|  |  |  | L_10d |

**Supplementary Figure 1e**

1. Cluster 1


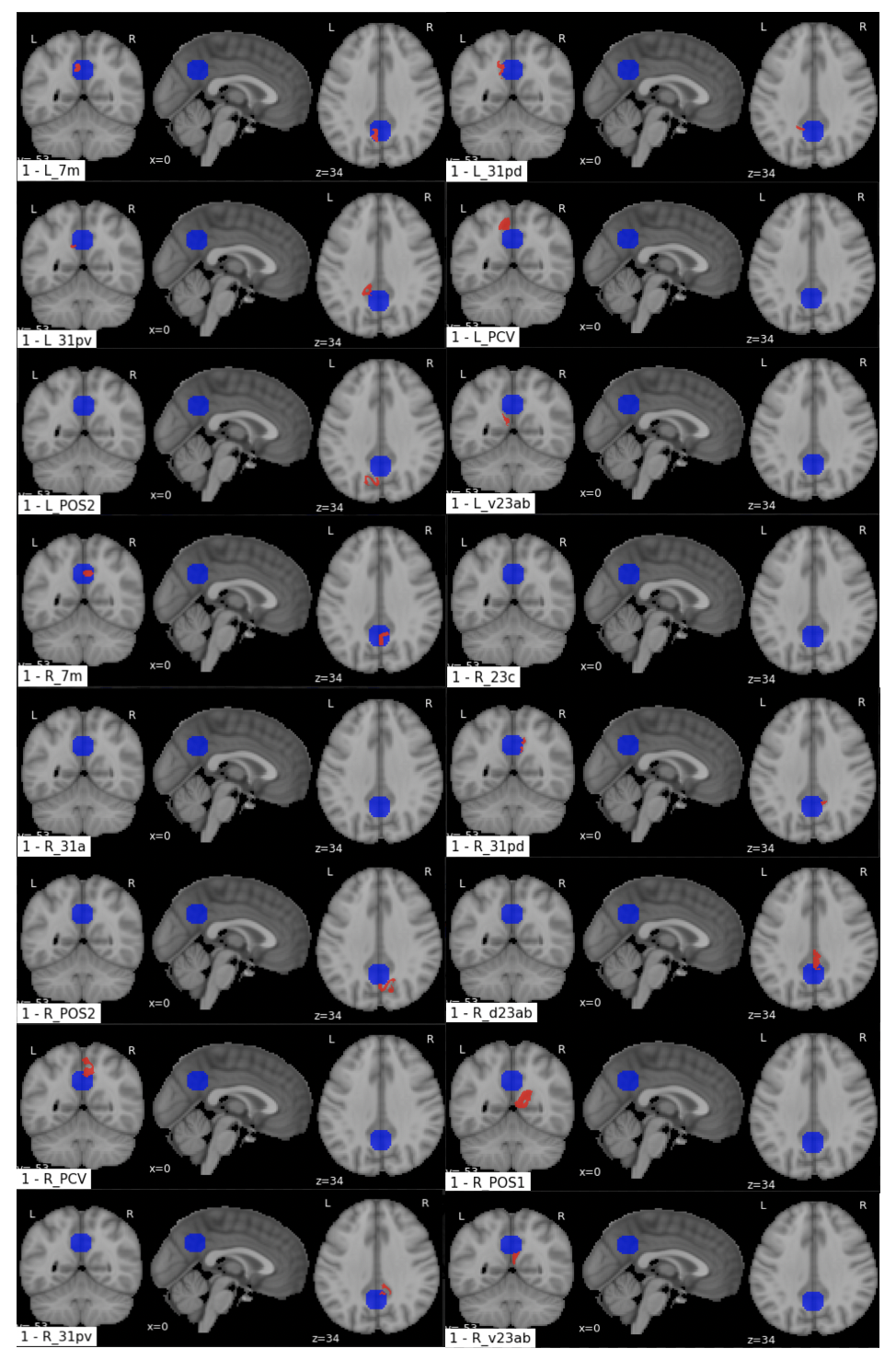


**Supplementary Figure 1e**

1. Cluster 2


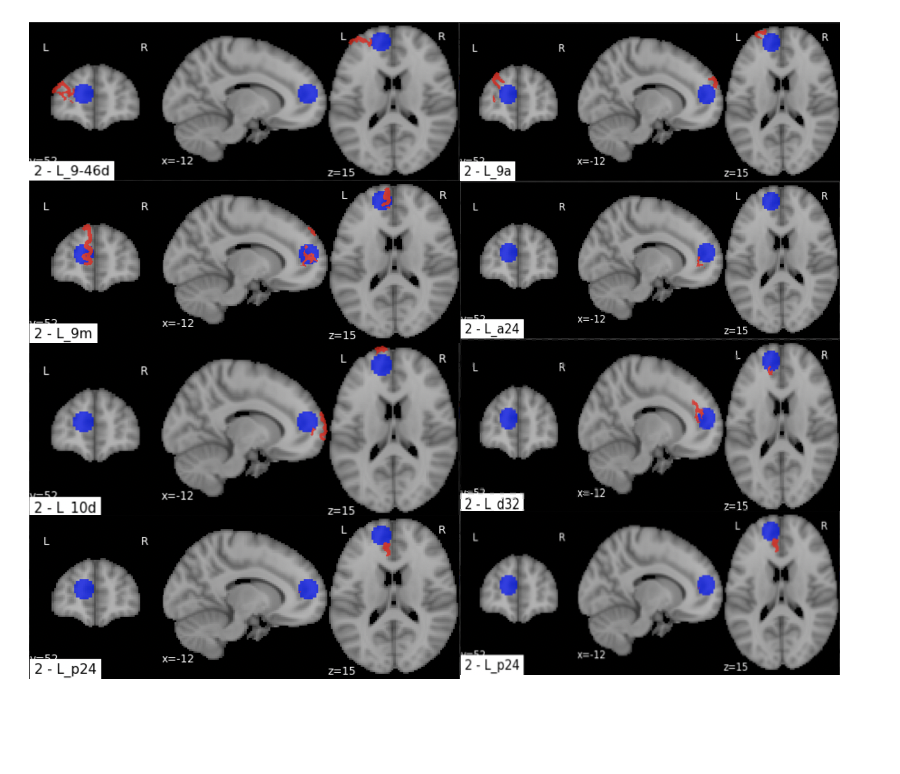


|  | **Cluster ID** | **MNI Coordinates** | **Parcellation Name** |
| --- | --- | --- | --- |
|  | 1 | -0.4 -53.0 34.3 | R_7m |
|  |  |  | R_31pd |
|  |  |  | R_31pv |
|  |  |  | R_PCV |
|  |  |  | R_31a |
|  |  |  | R_POS2 |
|  |  |  | R_v23ab |
|  |  |  | R_d23ab |
|  |  |  | R_23c |
|  |  |  | R_POS1 |
|  |  |  | L_7m |
|  |  |  | L_PCV |
|  |  |  | L_POS2 |
|  |  |  | L_d23ab |
|  |  |  | L_v23ab |
|  |  |  | L_31pv |
|  |  |  | L_31pd |
|  | 2 | -12.9 52.8 15.5 | L_9m |
|  |  |  | L_p24 |
|  |  |  | L_d32 |
|  |  |  | L_10d |
|  |  |  | L_a24 |
|  |  |  | L_p32 |
|  |  |  | L_9a |

**Supplementary Figure 1e**

1. Table corresponding to the 2 clusters for: **Neuropathic HC > CPP _ALE**

**Supplementary Figure 1f**

1. Cluster 1


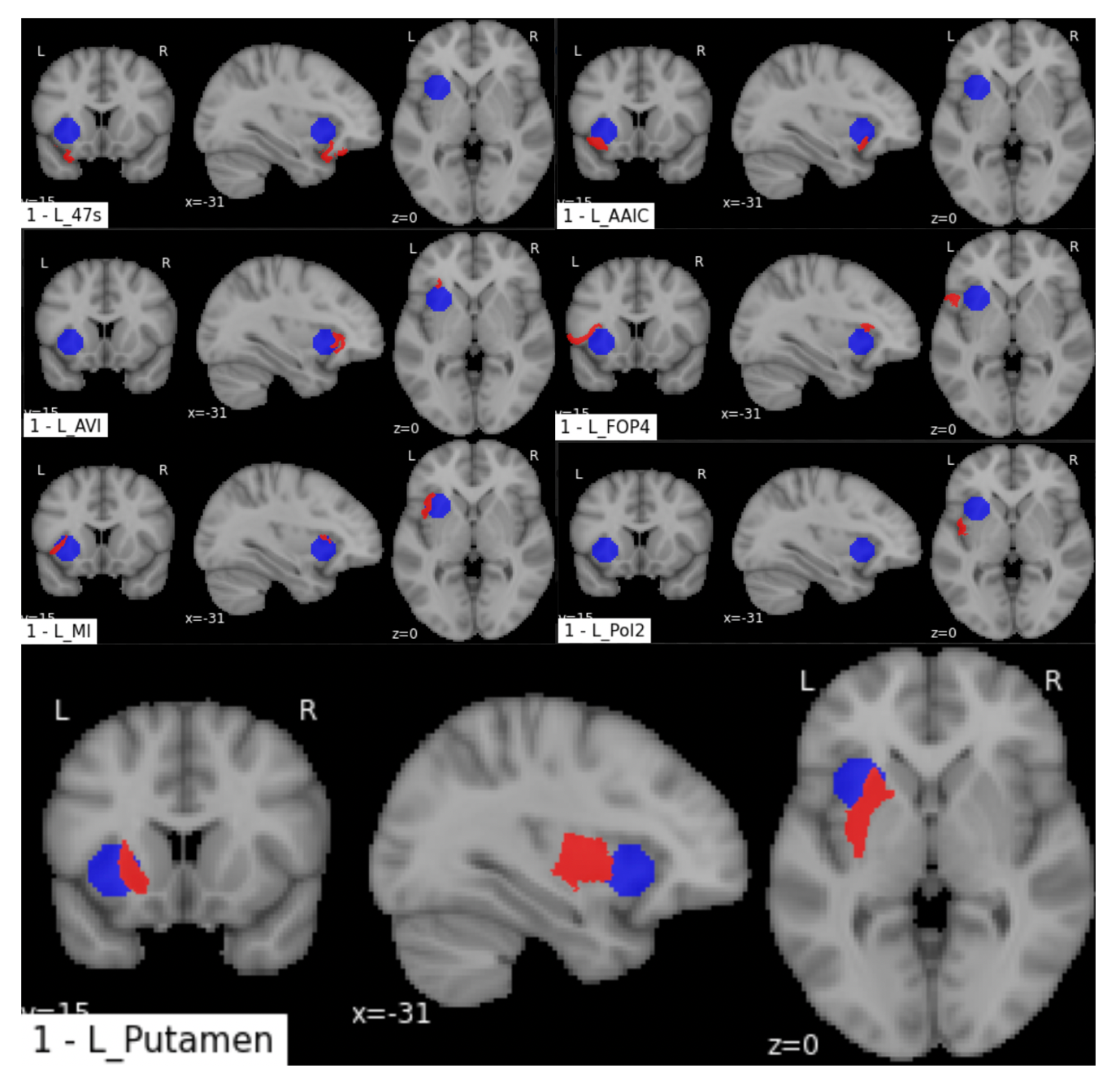


**Supplementary Figure 1f**

1. Cluster 2


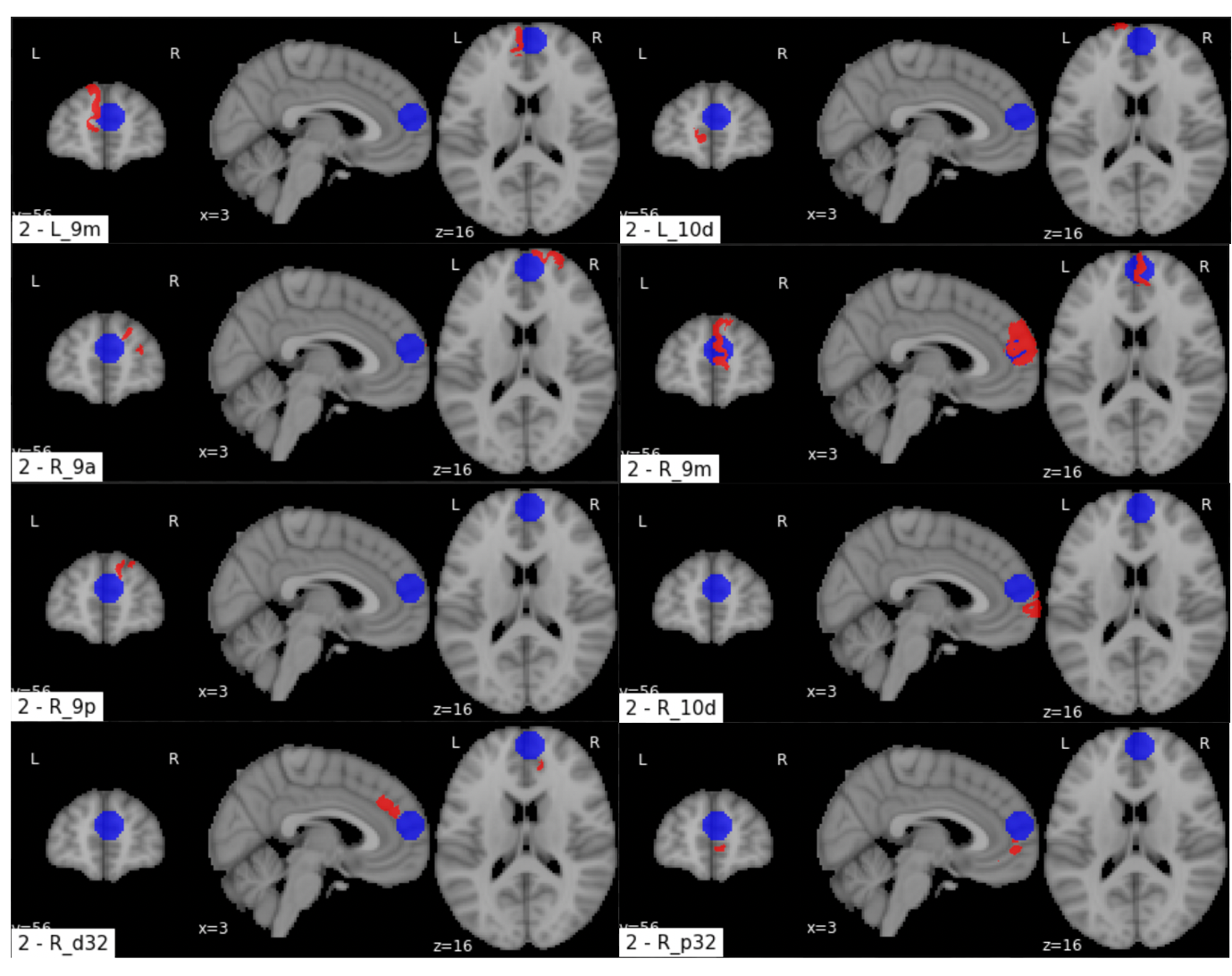


**Supplementary Figure 1f**

1. Cluster 3


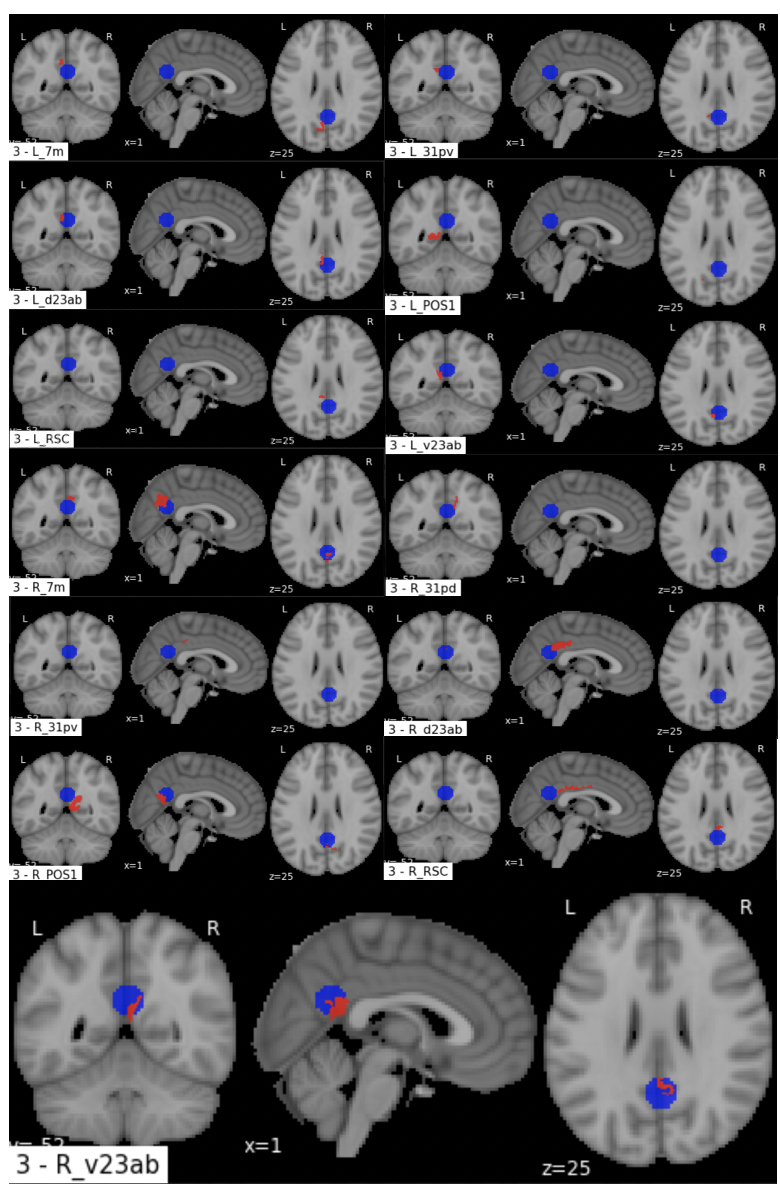


**Supplementary Figure 1f**

1. Table corresponding to the 3 clusters for: **Nociplastic HC > CPP _ALE**

|  | **Cluster ID** | **MNI Coordinates** | **Parcellation Name** |
| --- | --- | --- | --- |
|  | 1 | -31.5, 15.0, -0.2 | L_Putamen |
|  |  |  | L_AAIC |
|  |  |  | L_47s |
|  |  |  | L_AVI |
|  |  |  | L_MI |
|  |  |  | L_FOP4 |
|  |  |  | L_PoI2 |
|  | 2 | 3.8, 56.7 ,16.4 | R_9a |
|  |  |  | R_9p |
|  |  |  | R_9m |
|  |  |  | R_10d |
|  |  |  | R_p32 |
|  |  |  | R_d32 |
|  |  |  | L_9m |
|  |  |  | L_10d |
|  | 3 | 1.8 -52.9 25.7 | R_31pd |
|  |  |  | R_31pv |
|  |  |  | R_POS1 |
|  |  |  | R_v23ab |
|  |  |  | R_7m |
|  |  |  | R_d23ab |
|  |  |  | R_RSC |
|  |  |  | L_7m |
|  |  |  | L_d23ab |
|  |  |  | L_v23ab |
|  |  |  | L_POS1 |
|  |  |  | L_RSC |
|  |  |  | L_31pv |

**Supplementary Figure 1g**

1. Cluster 1


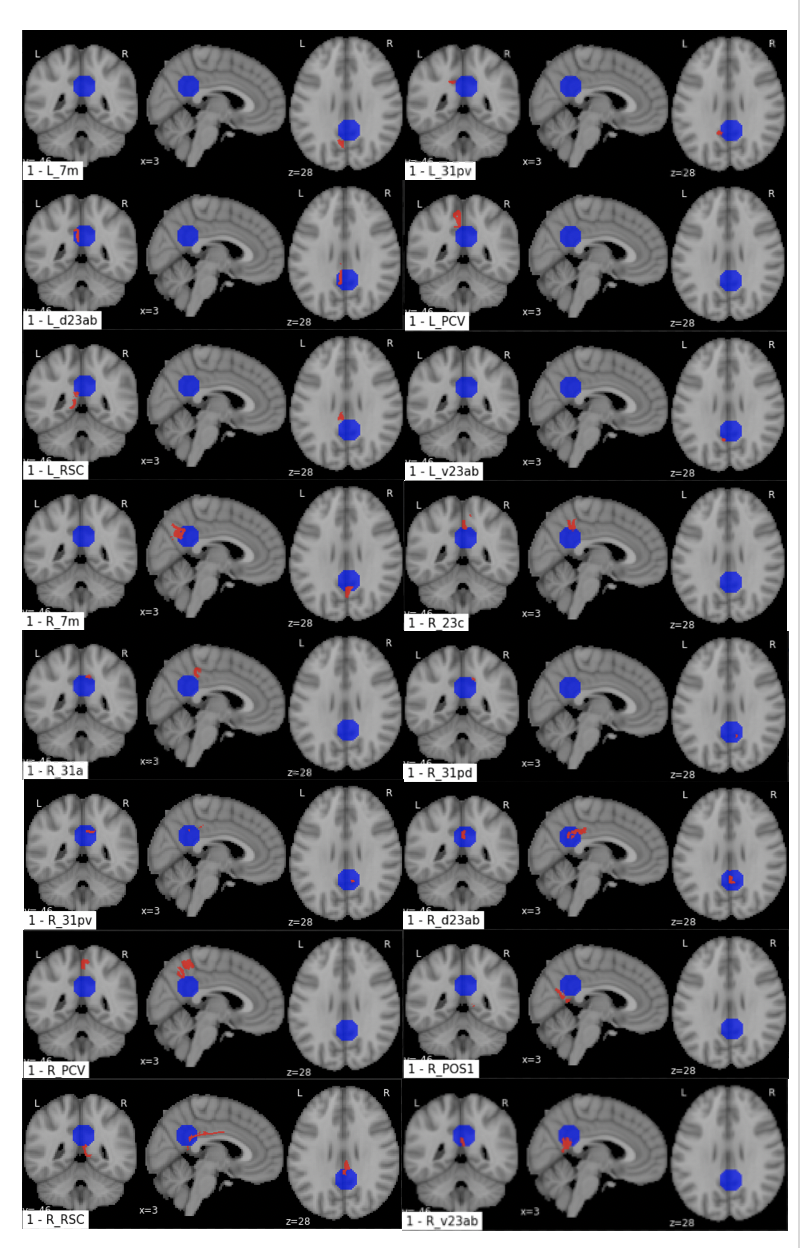


**Supplementary Figure 1g**

|  | **Cluster ID** | **MNI Coordinates** | **Parcellation Name** |
| --- | --- | --- | --- |
|  | 1 | 3.7, -46.4, 28.4 | R_31pd |
|  |  |  | R_31pv |
|  |  |  | R_POS1 |
|  |  |  | R_31a |
|  |  |  | R_7m |
|  |  |  | R_v23ab |
|  |  |  | R_RSC |
|  |  |  | R_PCV |
|  |  |  | R_d23ab |
|  |  |  | R_23c |
|  |  |  | L_PCV |
|  |  |  | L_7m |
|  |  |  | L_d23ab |
|  |  |  | L_v23ab |
|  |  |  | L_RSC |
|  |  |  | L_31pv |

Table corresponding to the 1 cluster for: **Nociceptive HC > CPP _ALE**

**Supplementary Figure 2:**


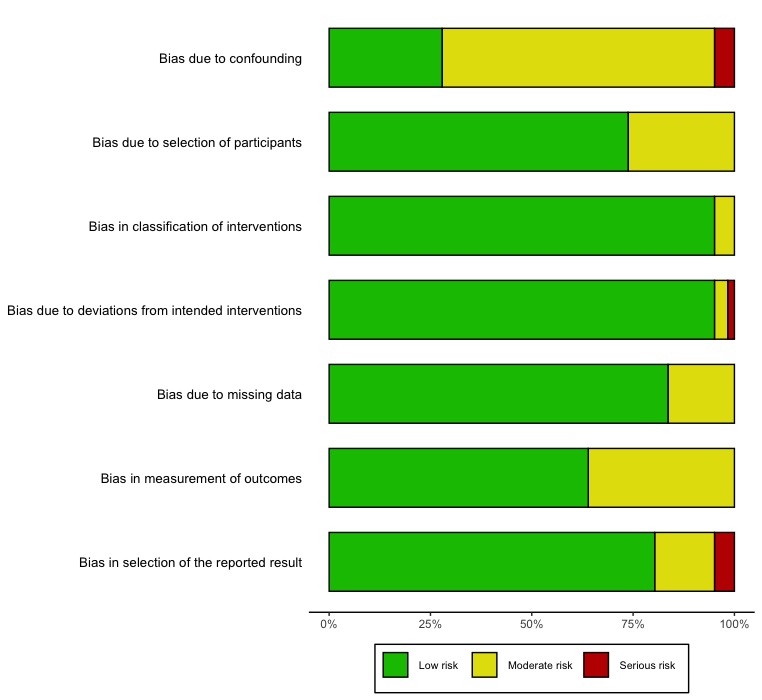


**Supplementary References**

1. Lim M, Nascimento TD, Kim DJ, Ellingrod VL, DaSilva AF. Aberrant Brain Signal Variability and COMT Genotype in Chronic TMD Patients. *Journal of Dental Research*. 2021;100(7):714. doi:10.1177/0022034521994089
2. Lim M, Jassar H, Kim DJ, Nascimento TD, DaSilva AF. Differential alteration of fMRI signal variability in the ascending trigeminal somatosensory and pain modulatory pathways in migraine. *The Journal of Headache and Pain*. 2021;22(1):4. doi:10.1186/s10194-020-01210-6
3. Barroso J, Wakaizumi K, Reis AM, et al. Reorganization of functional brain network architecture in chronic osteoarthritis pain. *Human Brain Mapping*. 2020;42(4):1206. doi:10.1002/hbm.25287
4. Huang J, Li Y, Xie H, et al. Abnormal Intrinsic Brain Activity and Neuroimaging-Based fMRI Classification in Patients With Herpes Zoster and Postherpetic Neuralgia. *Front Neurol*. 2020;11:532110. doi:10.3389/fneur.2020.532110
5. Park E, Cha H, Kim E, et al. Alterations in power spectral density in motor- and pain-related networks on neuropathic pain after spinal cord injury. *NeuroImage : Clinical*. 2020;28:102342. doi:10.1016/j.nicl.2020.102342
6. Liu Q, Liao Z, Zhang Y, et al. Pain- and Fatigue-Related Functional and Structural Changes in Ankylosing Spondylitis: An fRMI Study. *Frontiers in Medicine*. 2020;7:193. doi:10.3389/fmed.2020.00193
7. Yue X, Du Y. Altered intrinsic brain activity and regional cerebral blood flow in patients with chronic neck and shoulder pain. *Polish Journal of Radiology*. 2020;85:e155. doi:10.5114/pjr.2020.94063
8. Dai H, Jiang C, Wu G, et al. A combined DTI and resting state functional MRI study in patients with postherpetic neuralgia. *Jpn J Radiol*. 2020;38(5):440-450. doi:10.1007/s11604-020-00926-4
9. Li JL, Yan CQ, Wang X, et al. Brain Functional Alternations of the Pain-related Emotional and Cognitive Regions in Patients with Chronic Shoulder Pain. *Journal of Pain Research*. 2020;13:575-583. doi:10.2147/JPR.S220370
10. Zhang Y, Zhu Y, Pei Y, et al. Disrupted interhemispheric functional coordination in patients with chronic low back-related leg pain: a multiscale frequency-related homotopic connectivity study. *J Pain Res*. 2019;12:2615-2626. doi:10.2147/JPR.S213526
11. Yan J, Li M, Fu S, et al. Alterations of Dynamic Regional Homogeneity in Trigeminal Neuralgia: A Resting-State fMRI Study. *Frontiers in Neurology*. 2019;10:1083. doi:10.3389/fneur.2019.01083
12. Zhang Y, Mao Z, Pan L, et al. Frequency-specific alterations in cortical rhythms and functional connectivity in trigeminal neuralgia. *Brain Imaging Behav*. 2019;13(6):1497-1509. doi:10.1007/s11682-019-00105-8
13. Kaplan CM, Schrepf A, Vatansever D, et al. Functional and neurochemical disruptions of brain hub topology in chronic pain. *Pain*. 2019;160(4):973-983. doi:10.1097/j.pain.0000000000001480
14. Gu L, Hong S, Jiang J, et al. Bidirectional alterations in ALFF across slow-5 and slow-4 frequencies in the brains of postherpetic neuralgia patients. *Journal of Pain Research*. 2018;12:39. doi:10.2147/JPR.S179077
15. Rogachov A, Cheng JC, Hemington KS, et al. Abnormal Low-Frequency Oscillations Reflect Trait-Like Pain Ratings in Chronic Pain Patients Revealed through a Machine Learning Approach. *J Neurosci*. 2018;38(33):7293-7302. doi:10.1523/JNEUROSCI.0578-18.2018
16. Yuan J, Cao S, Huang Y, et al. Altered Spontaneous Brain Activity in Patients With Idiopathic Trigeminal Neuralgia: A Resting-state Functional MRI Study. *Clin J Pain*. 2018;34(7):600-609. doi:10.1097/AJP.0000000000000578
17. Hong S, Gu L, Zhou F, et al. Altered functional connectivity density in patients with herpes zoster and postherpetic neuralgia. *Journal of Pain Research*. 2018;11:881. doi:10.2147/JPR.S154314
18. Cao S, Li Y, Deng W, et al. Local Brain Activity Differences Between Herpes Zoster and Postherpetic Neuralgia Patients: A Resting-State Functional MRI Study. *Pain Physician*. 2017;20(5):E687-E699.
19. Cao S, Song G, Zhang Y, et al. Abnormal Local Brain Activity Beyond the Pain Matrix in Postherpetic Neuralgia Patients: A Resting-State Functional MRI Study. *Pain Physician*. 2017;20(2):E303-E314.
20. Yu CX, Ji TT, Song H, et al. Abnormality of spontaneous brain activities in patients with chronic neck and shoulder pain: A resting-state fMRI study. *J Int Med Res*. 2017;45(1):182-192. doi:10.1177/0300060516679345
21. Wang Y, Zhang X, Guan Q, Wan L, Yi Y, Liu CF. Altered regional homogeneity of spontaneous brain activity in idiopathic trigeminal neuralgia. *Neuropsychiatr Dis Treat*. 2015;11:2659-2666. doi:10.2147/NDT.S94877
22. He SS, Li F, Song F, et al. Spontaneous neural activity alterations in temporomandibular disorders: a cross-sectional and longitudinal resting-state functional magnetic resonance imaging study. *Neuroscience*. 2014;278:1-10. doi:10.1016/j.neuroscience.2014.07.067
23. Zou Y, Tang W, Qiao X, Li J. Aberrant modulations of static functional connectivity and dynamic functional network connectivity in chronic migraine. *Quant Imaging Med Surg*. 2021;11(6):2253-2264. doi:10.21037/qims-20-588
24. Li Z, Zhou J, Cheng S, et al. Cerebral fractional amplitude of low-frequency fluctuations may predict headache intensity improvement following acupuncture treatment in migraine patients. *J Tradit Chin Med*. 2020;40(6):1041-1051. doi:10.19852/j.cnki.jtcm.2020.06.016
25. Lan F, Lin G, Cao G, et al. Altered Intrinsic Brain Activity and Functional Connectivity Before and After Knee Arthroplasty in the Elderly: A Resting-State fMRI Study. *Front Neurol*. 2020;11:556028. doi:10.3389/fneur.2020.556028
26. Zhao Z, Huang T, Tang C, et al. Altered resting-state intra- and inter- network functional connectivity in patients with persistent somatoform pain disorder. *PLoS One*. 2017;12(4):e0176494. doi:10.1371/journal.pone.0176494
27. Wang Y, Xu C, Zhai L, et al. Spatial–temporal signature of resting-state BOLD signals in classic trigeminal neuralgia. *Journal of Pain Research*. 2017;10:2741. doi:10.2147/JPR.S143734
28. Huang T, Zhao Z, Yan C, et al. Altered Spontaneous Activity in Patients with Persistent Somatoform Pain Disorder Revealed by Regional Homogeneity. *PLoS One*. 2016;11(3):e0151360. doi:10.1371/journal.pone.0151360
29. Pujol J, Macià D, Garcia-Fontanals A, et al. The contribution of sensory system functional connectivity reduction to clinical pain in fibromyalgia. *Pain*. 2014;155(8):1492-1503. doi:10.1016/j.pain.2014.04.028
30. Ihara N, Wakaizumi K, Nishimura D, et al. Aberrant resting-state functional connectivity of the dorsolateral prefrontal cortex to the anterior insula and its association with fear avoidance belief in chronic neck pain patients. *PLoS ONE*. 2019;14(8):e0221023. doi:10.1371/journal.pone.0221023
31. Liao Q, Hu R, Huang D, Yan X, Guo G, Li X. Abnormal baseline brain activity in patients with cancer-induced bone pain: A resting-state functional magnetic resonance imaging study. 2016;9:14499-14506.
32. Kolesar TA, Bilevicius E, Kornelsen J. Salience, central executive, and sensorimotor network functional connectivity alterations in failed back surgery syndrome. *Scand J Pain*. 2017;16:10-14. doi:10.1016/j.sjpain.2017.01.008
33. Lu YC, Zhang H, Zheng MX, et al. Local and Extensive Neuroplasticity in Carpal Tunnel Syndrome: A Resting-State fMRI Study. *Neurorehabil Neural Repair*. 2017;31(10-11):898-909. doi:10.1177/1545968317723749
34. Hodkinson DJ, Wilcox SL, Veggeberg R, et al. Increased Amplitude of Thalamocortical Low-Frequency Oscillations in Patients with Migraine. *J Neurosci*. 2016;36(30):8026-8036. doi:10.1523/JNEUROSCI.1038-16.2016
35. Ma X, Li S, Tian J, et al. Altered brain spontaneous activity and connectivity network in irritable bowel syndrome patients: A resting-state fMRI study. *Clin Neurophysiol*. 2015;126(6):1190-1197. doi:10.1016/j.clinph.2014.10.004
36. Li Z, Zeng F, Yin T, et al. Acupuncture modulates the abnormal brainstem activity in migraine without aura patients. *Neuroimage Clin*. 2017;15:367-375. doi:10.1016/j.nicl.2017.05.013
37. Chen J, Wang Z, Tu Y, et al. Regional Homogeneity and Multivariate Pattern Analysis of Cervical Spondylosis Neck Pain and the Modulation Effect of Treatment. *Frontiers in Neuroscience*. 2018;12:900. doi:10.3389/fnins.2018.00900
38. Zhang J, Su J, Wang M, et al. The sensorimotor network dysfunction in migraineurs without aura: a resting-state fMRI study. *J Neurol*. 2017;264(4):654-663. doi:10.1007/s00415-017-8404-4
39. Li C, Wei X, Zou Q, et al. Cerebral functional deficits in patients with ankylosing spondylitis- an fMRI study. *Brain Imaging Behav*. 2017;11(4):936-942. doi:10.1007/s11682-016-9565-y
40. Gao Q, Xu F, Jiang C, et al. Decreased functional connectivity density in pain-related brain regions of female migraine patients without aura. *Brain Res*. 2016;1632:73-81. doi:10.1016/j.brainres.2015.12.007
41. Ao W, Cheng Y, Chen M, et al. Intrinsic brain abnormalities of irritable bowel syndrome with diarrhea: a preliminary resting-state functional magnetic resonance imaging study. *BMC Med Imaging*. 2021;21(1):4. doi:10.1186/s12880-020-00541-9
42. Chou KH, Yang FC, Fuh JL, et al. Bout-associated intrinsic functional network changes in cluster headache: A longitudinal resting-state functional MRI study. *Cephalalgia*. 2017;37(12):1152-1163. doi:10.1177/0333102416668657
43. Chen Z, Chen X, Liu M, Dong Z, Ma L, Yu S. Altered functional connectivity architecture of the brain in medication overuse headache using resting state fMRI. *The Journal of Headache and Pain*. 2017;18(1):25. doi:10.1186/s10194-017-0735-0
44. Liu S, Luo S, Yan T, et al. Differential Modulating Effect of Acupuncture in Patients With Migraine Without Aura: A Resting Functional Magnetic Resonance Study. *Front Neurol*. 2021;12:680896. doi:10.3389/fneur.2021.680896
45. Zhang YN, Huo JW, Huang YR, Hao Y, Chen ZY. Altered amplitude of low-frequency fluctuation and regional cerebral blood flow in females with primary dysmenorrhea: a resting-state fMRI and arterial spin labeling study. *Journal of Pain Research*. 2019;12:1243. doi:10.2147/JPR.S177502
46. Zhou F, Gu L, Hong S, et al. Altered low-frequency oscillation amplitude of resting state-fMRI in patients with discogenic low-back and leg pain. *Journal of Pain Research*. 2018;11:165. doi:10.2147/JPR.S151562
47. Zhao L, Liu J, Dong X, et al. Alterations in regional homogeneity assessed by fMRI in patients with migraine without aura stratified by disease duration. *J Headache Pain*. 2013;14(1):85. doi:10.1186/1129-2377-14-85
48. Zhang Y, Li K shi, Liu H wei, et al. Acupuncture treatment modulates the resting-state functional connectivity of brain regions in migraine patients without aura. *Chin J Integr Med*. 2016;22(4):293-301. doi:10.1007/s11655-015-2042-4
49. Li Z, Zhou J, Lan L, et al. Concurrent brain structural and functional alterations in patients with migraine without aura: an fMRI study. *The Journal of Headache and Pain*. 2020;21(1):141. doi:10.1186/s10194-020-01203-5
50. Zhao L, Liu J, Yan X, et al. Abnormal brain activity changes in patients with migraine: a short-term longitudinal study. *J Clin Neurol*. 2014;10(3):229-235. doi:10.3988/jcn.2014.10.3.229
51. Wang P, Du H, Chen N, et al. Regional homogeneity abnormalities in patients with tension-type headache: a resting-state fMRI study. *Neurosci Bull*. 2014;30(6):949-955. doi:10.1007/s12264-013-1468-6
52. Wu TH, Tu CH, Chao HT, et al. Dynamic Changes of Functional Pain Connectome in Women with Primary Dysmenorrhea. *Sci Rep*. 2016;6(1):24543. doi:10.1038/srep24543
53. Zhou F, Wu L, Guo L, Zhang Y, Zeng X. Local connectivity of the resting brain connectome in patients with low back-related leg pain: A multiscale frequency-related Kendall’s coefficient of concordance and coherence-regional homogeneity study. *Neuroimage Clin*. 2019;21:101661. doi:10.1016/j.nicl.2019.101661
54. Chen XF, Guo Y, Lu XQ, et al. Aberrant Intraregional Brain Activity and Functional Connectivity in Patients With Diarrhea-Predominant Irritable Bowel Syndrome. *Front Neurosci*. 2021;15:721822. doi:10.3389/fnins.2021.721822
55. Ke J, Qi R, Liu C, et al. Abnormal regional homogeneity in patients with irritable bowel syndrome: A resting-state functional MRI study. *Neurogastroenterol Motil*. 2015;27(12):1796-1803. doi:10.1111/nmo.12692
56. Li L, Ma J, Xu JG, et al. Brain functional changes in patients with Crohn’s disease: A resting-state fMRI study. *Brain Behav*. 2021;11(8):e2243. doi:10.1002/brb3.2243
57. Jiang J, Gu L, Bao D, et al. Altered homotopic connectivity in postherpetic neuralgia: a resting state fMRI study. *Journal of Pain Research*. 2016;9:877. doi:10.2147/JPR.S117787
58. Qiu E chao, Yu S yuan, Liu R zhuo, Wang Y, Ma L, Tian L xia. Altered regional homogeneity in spontaneous cluster headache attacks: a resting-state functional magnetic resonance imaging study. *Chin Med J (Engl)*. 2012;125(4):705-709.
59. Chen C, Yan M, Yu Y, et al. Alterations in Regional Homogeneity Assessed by fMRI in Patients with Migraine Without Aura. *J Med Syst*. 2019;43(9):298. doi:10.1007/s10916-019-1425-z
60. Lin Y, Bai Y, Liu P, et al. Alterations in regional homogeneity of resting-state cerebral activity in patients with chronic prostatitis/chronic pelvic pain syndrome. *PLoS ONE*. 2017;12(9):e0184896. doi:10.1371/journal.pone.0184896
61. Zhang J, Xu T, Wang L, et al. Dynamic alterations of amplitude of low-frequency fluctuations in patients with chronic neck pain. *Psychoradiology*. 2021;1(3):110-117. doi:10.1093/psyrad/kkab011
